# Children’s rights and needs during war: the case of adolescents in Israel

**DOI:** 10.3389/fpsyg.2026.1719621

**Published:** 2026-03-02

**Authors:** Yonat Rum, Erez Milsthen, Heba F. Zedan, Tali Gal

**Affiliations:** 1 Seymour Fox School of Education, The Hebrew University of Jerusalem, Jerusalem, Israel; 2 Department of Psychology, The Hebrew University of Jerusalem, Jerusalem, Israel; 3 Child and Youth Rights Program, The Minerva Center for Human Rights, Faculty of Law, The Hebrew University of Jerusalem, Jerusalem, Israel

**Keywords:** adolescents, armed conflict, children’s needs, children’s rights, Convention on the Rights of the Child, CRC, terror, war

## Abstract

**Introduction:**

This study aims to examine the attainment of rights and needs of adolescents during the war following the October 7th 2023 terror attack on Israel. A normative analysis of the human rights of children and youth, based on the United Nations Convention on the Rights of the Child (CRC), was integrated with an empirical examination of children’s rights and needs attainment. Committed to a children’s rights epistemology, the empirical examination is based on children’s own reports. Additional aims were to explore whether gaps existed between the adolescents’ perspectives and those of their parents, and across demographic groups (using Hebrew and Arabic surveys).

**Methods:**

A novel questionnaire was developed based on key children’s rights detailed in the CRC and organized according to the “Three P’s” classification: protection (whether children are safe from harm), provision (continuity and equality in public services), and participation (children’s access to safe information, participation in decision-making, privacy, and freedom of expression). Data were collected in Hebrew from 359 adolescent-parent dyads between May and July 2025. In a second wave, data were collected in Hebrew and Arabic from additional samples of adolescents (*n* = 101; *n* = 24) between August and September 2025. A mixed-methods approach was employed to analyze the quantitative data, alongside qualitative analysis of text responses to open-ended questions.

**Results:**

Protection: while basic safety seems to be maintained for adolescents, exposure to violence, mainly verbal, in schools was noted. Among responders to the Arabic survey, higher percentages of verbal assault at home were reported. Fear of “the other” emerges as a significant theme in text responses. Provision: education continuity is challenged, with Arab schools showing more stability Participation: Arabic-speaking adolescents report feeling less free to express their opinions. Parents were reported as a central source of safe information for adolescents, mainly in the Hebrew surveys. Text responses highlight the need of some to “talk more” and “be heard more” despite their young age.

**Discussion:**

By employing an interdisciplinary framework that combines a normative rights-based analysis with an empirical examination of children’s reports on the attainment of their rights and needs, this study contributes to the broader understanding of how war impacts adolescents, emphasizing often-overlooked areas such as freedom of speech, equality, access to safe information, and participation. Findings highlight the impacts of prolonged war on the sense of safety beyond immediate needs.

## 1 Introduction

Exposure to war detrimentally impacts the lives of children and youth in various ways and domains. Examining impacts on mental health, literature shows that children and adolescents exposed to war, terrorism, or armed conflicts show an increase in post-traumatic symptoms and post-traumatic stress disorders (PTSD), elevated behavioral and emotional problems, difficulties with sleep and play, aggression, emotional numbing, and psychosomatic symptoms (Attanayake et al., 2009; Dimitry, 2012; Kadir et al., 2019; Slone and Mann, 2016). Research has also revealed the complex and interconnected nature of war’s impact on child development. Kadir et al. (2019) conducted a systematic review of 50 studies spanning 1990–2017, examining children in war-affected regions across Africa, Asia, Eastern Europe, and the Middle East. Their analysis demonstrates that psychological distress represents the most frequently documented outcome among war-affected children, and that these outcomes exhibit a clear dose–response relationship: the more cumulative the exposure to violence and instability, the greater the risk and severity of psychological symptoms. Werner’s (2012) comprehensive review of global studies also found that children exposed to armed conflict commonly exhibit elevated rates of PTSD, depression, and anxiety, and similarly pointed out that symptom severity correlates with the recency and intensity of exposure. It was also found that more early exposure to violence predicts more post-traumatic stress symptoms, as was shown in a longitudinal study surveying Palestinian and Israeli children exposed to protracted conflict (Huesmann et al., 2022). A qualitative thematic analysis of interviews with Palestinian youth and adults in East Jerusalem (*n* = 24 adolescents, *n* = 8 adult parents and professionals; Zedan, 2025) suggested that such traumatic stress might be reflected by the youth experiencing perpetual threat, hypervigilance, intergenerational transmission of stress, emotional suppression and helplessness, normalization of abnormality, and distrust in protective systems (Zedan, 2025).

The psychological impact of war on children extends beyond immediate trauma responses to encompass long-term developmental consequences. Longitudinal studies reveal the persistence of psychological effects, with research on children traumatized during World War II showing that early trauma was linked to chronic PTSD, depression, and physical illnesses decades later. Among Cambodian children who experienced war trauma, 50% showed PTSD symptoms during high school, with 35–38% continuing to experience symptoms 12 years later. In Israel, it has been shown that children living in an area exposed to prolonged rocket attacks from Gaza in Southern Israel experience greater developmental, emotional, and behavioral problems than those in central Israel who were less exposed to the rocket attacks (Feldman et al., 2013; Shechory Bitton and Laufer, 2018).

While this body of literature focuses on psychological outcome measures, mostly collected through parental reports or standard clinical assessments, the present study employs a novel, interdisciplinary approach combining the empirical approach with a theoretical international law framework to examine the impact of war on adolescents through the lens of children’s rights and needs, and by relying on the adolescents’ own reports. Specifically, the study examines how the ongoing multifront war in Israel, which began with the Hamas attack on October 7th, 2023, has affected the attainment of rights and needs of adolescents in Israel.

### 1.1 The war following the October 7th 2023 terror attack: impacts on children in Israel

During the Jewish holiday of Simchat Torah, on the morning of Saturday, October 7, 2023, the Hamas terrorist organization^2^ launched a surprise attack on the Israeli towns and villages bordering the Gaza Strip. In this attack, Palestinian Armed Groups breached the border into Israel through land, sea, and air, targeting military and civilian areas with indiscriminate rocket fire and massacring^3^ individuals and families on the streets, at a music festival, and in their homes (Human Rights Watch, 2024). When taking into account the number of victims per capita, it is considered to be one of the deadliest terrorist attacks in modern times (Statista.com, 2023). The Palestinian Armed Groups who broke into Israel also abducted over 200 Israeli and foreign citizens, including women, the elderly, adolescents, children, and toddlers (Human Rights Watch, 2024). The attacks extended to the north of Israel, where missiles were fired at civilian centers and Israeli Defense Forces’ positions from Lebanon by the Hezbollah terror group. Israel faced significant displacement of residents from affected areas: civilians living close to the border with Gaza, who had experienced the carnage firsthand, have been evacuated. Residents of the northern border areas have also been evacuated. Communities across Israel have been targeted by rocket attacks, so some individual families from other areas also had to leave their damaged homes and move to temporary accommodations (Ministry of Foreign Affairs, 2024; Sabag et al., 2024). These events marked the beginning of a war whose end is not yet in sight at the time of the writing of these lines. Additional fronts of armed conflict have opened since the war began, with the continuous firing of missiles on Israel by the Houthis terror group from Yemen and the 12-day Israel-Iran War in June 2025. In response, Israel has also launched military operations against armed groups and infrastructure across multiple fronts. These operations have resulted in widespread destruction and many thousands of civilian casualties in Gaza, including children and youth (Bessell and O’Sullivan, 2026).

It is already clear that this war is an unprecedented event in Israel’s history (see: Codish et al., 2024; Levi-Belz et al., 2024; Yehene et al., 2024), with profound and multifaceted impacts on children and adolescents. The initial October 7th attack resulted in the deaths of 56 infants, children, and youth under 18, with 39 children and toddlers abducted and taken from Israel into captivity in Gaza (National Council for the Child, 2024; Ziv et al., 2024; Bron-Harlev et al., 2025), some were abducted with their parents (Tsur et al., 2025) while others were separated from their parents and siblings during abduction or in captivity (see for example Knafo-Noam and Segal, 2025). The scale of family disruption is reflected in statistics showing that 359 Israeli children lost at least one parent to terrorist attacks, 615 lost a parent serving in Israeli security forces, and over 1,000 children lost siblings during the war, as of 22.7.24 (Ministry of Justice, Social Justice Division, 2024). Displacement is another challenge, with 253,000 people evacuated from border communities by November 2023, including approximately 66,000 children and youth (National Council for the Child, 2024). As of May 2024, 7,976 children remained displaced, residing in temporary accommodations (Committee on the Rights of the Child, 2024). Following the June 2025 Iranian missile attacks, thousands of families in Israel were displaced as homes were damaged or destroyed. The Knesset Information and Research Center (2024) identified multi-dimensional risks facing displaced children, including educational, emotional, developmental, and social challenges.

The Brookdale Institute report (Sabag et al., 2024) identified seven key domains of the war’s impact on children: physical health and development, emotional well-being, family affiliation, protection from others, learning and acquisition of skills, social belonging and community participation, and protection from self-endangering behaviors. Suggesting that the war’s psychological impact transcends geographical boundaries, the authors of this review conclude that apart from children who were directly exposed to carnage or have been evacuated from their homes, the war affects all children and youth in Israel. The sources of information for this review included policy documents, information sheets, and protocols of meetings of government forums and organizations that provide for the needs of families and children; literature on previous conflicts in Israel and worldwide and their effect on children, and the etiology of risk factors among children and youth as identified in the literature (Sabag et al., 2024).

Notably, a population uniquely impacted by the war is the Arab and Palestinian population in Israel, which consists of Muslim, Christian, Druze, and Bedouin communities – altogether about 21% of the Israeli population, and ~360,000 residents in East Jerusalem (Israel Democracy Institute, 2023; Halabi and Shoshana, 2024). These minorities face numerous gaps in socio-economic conditions compared with the Jewish majority, including lower educational achievements, higher poverty rates, reduced health indicators, and longstanding under-investment in the infrastructure of their towns, villages, and neighborhoods, which research shows account for a substantial share of the disparities (Daoud et al., 2018). In the context of the war, not only socio-economic issues and identity put these minorities in the face of unique challenges, but sometimes also direct family relations to relatives in Gaza who experience the severe impact of war there. Thus, their experiences of the war within the broader context of the Israeli-Palestinian conflict have unique complexity. A previous study by two of the current authors (HFZ and TG) involving Palestinian adolescents from East Jerusalem found that participants described a persistent sense of political persecution, including recurrent encounters with the Israeli forces, surveillance, and harassment in public spaces, and perceived restrictions on freedom of expression (Zedan and Gal, 2025; Zedan, 2025). In addition, many Arab towns and villages, specifically in the Bedouin residential areas, lack adequate protective infrastructure (e.g., shelters or “safe rooms”) from missile attacks (Al-Said and Braun-Lewensohn, 2024). In the present study, we thus aimed to ensure the representation of these minorities when examining the attainment of rights and needs of adolescents in Israel during war.

The impact of war on children and youth’s mental health has been documented across Israel, with increases in 2024 in anxiety, somatization, and depression - rates substantially higher than those reported in previous years, including during the COVID-19 pandemic (National Council for the Child, 2025). These statistics suggest that the psychological impact of war on children and youth extends beyond areas of direct physical exposure. Vulnerable populations were documented to experience even higher negative impact. For example, a study elucidating the experiences of families of autistic and non-autistic children in Israel found that in the 30 days following the 7 October 2023 terror attack, parents reported that their children (autistic and non-autistic) showed clinically significant posttraumatic stress symptoms, with autistic children reported to be experiencing even more posttraumatic stress (Rozenblat et al., 2024).

Thus, research conducted in Israel regarding the impact of the ongoing war on children and adolescents aligns with previous findings from around the world in highlighting the detrimental effects of terror and war, focusing mainly on mental health (Kadir et al., 2019). It also, similarly to much of the global literature, relied mainly on data collected from government and other organizations (e.g., Sabag et al., 2024), as well as clinical assessments or parents’ reports on themselves and their children (e.g., Gur et al., 2025; Rozenblat et al., 2024). It is more challenging to find studies that rely on children or adolescent participants, who report their own experiences and express their perspectives. Zedan and Gal (2025; reviewed above) attempted to do so in participatory research with Palestinian youth from East Jerusalem. However, this kind of qualitative work is, by nature, comprised of only a few participants. Another attempt to directly seek the perspective of youth in Israel during the war was made in 2024 by Kosher and colleagues.

The Haruv Institute report (Kosher et al., 2024) presents a study examining Israeli children’s lived experiences regarding their social relationships during the war, resources for coping, and what the children would like policymakers to know about the impact of war on them. Through conducting six focus groups encompassing a total of 51 children aged 10–17 years from Israeli communities in the Gaza-surrounding area, the researchers collected data between December 2023 and February 2024. Participants discussed the impact of war on their social relationships, and the content of the conversations was analyzed in a qualitative approach. The study also included distributing questionnaires to measure the participants’ social relationships before and after the October 7th carnage. The results emphasized complex and varied difficulties that extend beyond direct trauma exposure, as well as a variety of coping strategies. For example, while some children described loss of contact with friends due to displacement, others described intense (and sometimes too intense) social relationships while living with peers in the same temporary accommodation. Although children were not directly asked about school and learning, this theme emerged from their own choice of subjects to discuss, suggesting a lack of motivation, feelings of disengagement from school, and the feeling that some teachers do not understand them. Other reported challenges included a sense of privacy loss, desire to go back home alongside fear, experiences of sadness, stress, and anxiety.

Many children participating in the Haruv report focus groups described an experience of “invisibility”: the feeling that adults do not understand them and are not available to them, while also noticing that adults in their lives are experiencing profound difficulties, trauma, and dysfunction. Some children expressed the feeling that there was no place for their voices to be heard (Kosher et al., 2024). This report employed the theoretical prism of children’s rights, according to which a child should be viewed as a whole person and a subject in their own right. Thus, the inclusion of children as participants was not just a methodological choice, but also a conceptual and epistemological one. In the present study, we employed the same children’s rights theoretical framework, aiming to collect data on a larger scale from children across the country, and with a broader scope of children’s rights.

### 1.2 Children’s rights during war: a rights-based approach

A rights-based approach to studying the impact of armed conflicts on children provides a normative framework that recognizes children not merely as passive victims in need of protection, but as active rights-holders entitled to dignity, agency, and accountability from duty-bearers. Grounded in the UN Convention on the Rights of the Child (CRC), this perspective shifts the focus from needs-based interventions to legally and morally binding obligations that states and institutions must uphold. Goldhagen et al. (2019) argue that this approach reframes the problem of children in war as a systemic failure of international commitments, thereby elevating the ethical and legal imperatives for action. It emphasizes that violations of children’s rights – such as deprivation of education, loss of family care, forced displacement, and trauma – are not just humanitarian concerns, but breaches of codified international standards. Furthermore, a rights-based approach broadens the scope of investigating the impact of war on children beyond questions about their safety, and physical and mental health, to include concepts such as privacy, equality, freedom of expression, and participation in decision-making – all of which are internationally recognized human rights of children and youth. At the epistemological level, a rights-based approach recognizes that, despite their dependence on adults, children have interests, needs, and perspectives that are distinct from those of the adults surrounding them. Accordingly, a rights-based methodology is based on asking children directly about their experiences and needs. Thus, a children’s rights framework expands the current discourse about children in wars in three levels: it provides a broader spectrum of questions, promotes a child-centered methodology, and offers a robust foundation for advocacy, accountability, and sustained policy change.

The present study rests on the normative foundation of the CRC, which has achieved near-universal ratification and establishes clear legal obligations for the protection of children’s rights within member-state jurisdictions (Goldhagen et al., 2019). The CRC establishes over 20 children’s rights, which can be categorized into three domains: 1. Protection – protective rights against harm; 2. Provision – social rights ensuring access to services and resources; and 3. Participation – participatory rights involving children’s agency and engagement in family, community, and public life (Qvortrup, 1996; Okorodudu, 1998; Goldhagen et al., 2019; Freeman, 2020). The rights outlined in the CRC are interconnected in that the fulfillment of one right category often depends on the realization of others (Gal, 2011). For instance, the right to education serves not only as a provision right but also as a crucial protective mechanism that provides structure, normalcy, and psychosocial support (Franczak and Lutz, 2024). The CRC’s framework is also holistic, requiring each right to be applied with the principles of equality, the best interest of the child, and child development and participation in mind.

The human rights framework created by the CRC is also interdisciplinary in nature, integrating legal and psycho-social knowledge. By specifying broad terms such as development and the child’s best interest, it invites findings and insights from the social sciences about children’s needs to inform policymakers on ways to fill these general human rights principles with evidence-informed content (Gal, 2011). For example, in considering the right to development and its attainment during an armed conflict, one needs to explore developmental psychology knowledge regarding the required conditions for healthy development and how such conditions, such as stability, predictability, and supportive relationships, are fundamentally disrupted during warfare (Werner, 2012; Machel, 1996).

While numerous researchers studying the impact of armed conflict on children have framed their findings using a children’s rights lens (e.g., Kosher et al., 2024; Zedan and Gal, 2025), no comprehensive investigation yet captures how war undermines the entire spectrum of children’s rights envisioned by the CRC. The Machel Report (1996) was groundbreaking in reframing children affected by war as rights-holders, shifting international attention toward their protection and participation. However, it did not explicitly use the CRC as a blueprint for setting the research questions, leaving rights such as equality, freedom of expression, and privacy underexplored. Using the Machel Report’s evidence on killings, injuries, forced recruitment, displacement, and attacks on schools, Okorodudu (1998) mapped these findings onto the CRC framework, demonstrating how wars undermine children’s rights to survival, protection, development, and participation, including threats to basic rights to life, health, education, and free expression. Likewise, Goldhagen et al. (2019) proposed a comprehensive rights-based framework, linking armed conflict to health outcomes and calling for collaboration across agencies to protect children’s rights. Yet, both were primarily conceptual and did not generate new empirical data. Where empirical work exists, it tends to focus on single rights, especially education. For example, a UNESCO (2011) report documented school closures, teacher displacement, budgetary diversions, and destruction of infrastructure as central obstacles to learning and psychosocial well-being in conflict zones. Research on children’s participation rights during war has been extremely scarce, despite the centrality of the participation right in the CRC. One such project is Singh (2023) qualitative study in Chhattisgarh, India, which found that decisions regarding rehabilitation and institutional placement of conflict-affected children were typically made without children’s involvement. Thus, there is a scarcity of research that systematically applies the full spectrum of children’s rights to examine the realities of children in war.

### 1.3 The present study

The present study adopts an integrative theoretical framework that combines the normative rights-based approach established in the CRC with an empirical examination of children’s needs (Goldhagen et al., 2019; Kadir et al., 2019). A needs-only discourse is an objective assessment of what people need for a minimally decent or flourishing human life. A rights-only discourse is someone’s claim about their entitlements, typically expressed in their own voice. The integration of both children’s rights and children’s needs perspectives creates a more robust framework that bridges normative assertions regarding state obligations with empirical insights regarding children’s needs and perspectives (Franczak and Lutz, 2024; Gal, 2011). This dual approach also recognizes that while rights-based discourse effectively establishes legal and moral obligations, it must be complemented by an evidence-based understanding of children’s needs (Gal, 2011; Waldron, 2000). An even more robust framework is one that relies on universal consensus of children’s entitlements to seek their own perspectives on the level of attainment of their needs and rights, while also collecting parents’ reports, to provide triangulation and validation. Another reason for seeking adolescents’ self-reports in the present study is that some rights violations may not be observable to parents or captured in institutional records, for example, internal experiences and subjective aspects of violations of freedom of speech or the right to participation. Accordingly, the primary objective of the present study was to assess the extent to which adolescents’ rights and needs were fulfilled in Israel during war, according to their own reports and those of their parents. An additional objective was to explore whether there were gaps between the adolescents’ perspectives and those of their parents. Finally, we aimed to examine whether there were differences across demographic groups and levels of exposure to the war’s impact, both in terms of displacement and over time.

## 2 Materials and methods

The procedure consisted of developing a novel questionnaire, conducting a pilot study, and collecting data in two waves.

### 2.1 Participants

In the first wave, data were collected from 359 adolescents aged 14–18 years and their parents. In a second wave, data were collected from an additional sample of 101 Hebrew-speaking and 24 Arabic-speaking adolescents. The demographic characteristics of the three samples are detailed in Table 1.

**Table 1:** Demographic characteristics of the three samples of participants.

		Wave I	Wave II	
		Hebrew questionnaire *q*	Hebrew questionnaire *q*	Arabic questionnaire *q*
Children	*N*	349	101	24
	Age (split)	2.3% Grade 8 – Under 14 22.2% 14 years old 31.7% 15 years old 20.2% 16 years old 15.6% 17 years old 8.1% 18 years old	3% Grade 8 – Under 14 15.8% 14 years old 27.7% 15 years old 26.7% 16 years old 19.8% 17 years old 6.9% 18 years old	10% Grade 8 – Under 14 25% 14 years old 10% 15 years old 30% 16 years old 25% 17 years old
	% Girls	51.3%	53%	54.1%
Parents	*N*	359	–	–
	% Mothers	63.7%	–	–
Family	% Displaced families	8.1%	3%	0%
	Geographical area of residence	25.9% Central District 19.8% Southern District 18.1% Northern District 14.7% Haifa District 10.9% Tel Aviv District 10.6% Jerusalem District	46.5% Central District 14.9% Tel Aviv District 11.9% Southern District 10.9% Northern District 8.9% Haifa District 6.9% Jerusalem District	40% Central District 20% Southern District 20% Northern District 16% Haifa District 4% Jerusalem District
	Religion	92% Jewish 4.5% Muslim 1% Druze 0.5% Christian 2% no religion or chose not to answer	98% Jewish 1% Muslim 1% prefer not to answer	96% Muslim 4% Christian *(1 participant)
	Spoken language at home	89.9% Hebrew 5.7% Arabic 2.7% Russian 0.8% English 0.5% Yiddish 0.2% other	95% Hebrew 2% Russian 2% English 1% other	100% Arabic

Gender data was missing for 2 parents in the sample.

### 2.2 Procedure

#### 2.2.1 Questionnaire development

The questionnaire was developed to reflect the CRC’s normative framework of children’s rights, which are founded on children’s universal needs, spanning the domains of protection, provision, and participation and agency, but instead of using legal language, it used day-to-day needs terminology. First, the relevant CRC rights were identified (Protection – Articles 3 [best interests], 19 [protection from violence], 37 and 40 [protection from torture and cruel punishment; due process]; Provision – Articles 2 [equality], 6, 26, 27 [development, adequate living conditions, and social security], 24 and 39 [health, healthy environment, rehabilitation], and 28, 29, 31 [education, leisure, and social activities]; Participation – Articles 12 [expressing views], 13–15 [freedom of speech, thought, conscience, religion, and association], 16 [privacy], 17 [access to safe media], and 30 [culture]). Then, the specific obligations towards children that each article creates in the context of war were articulated (for example, Articles 13, 14, and 15 were translated into a more concrete construct of “guaranteeing freedom of expression and preventing hate speech and violent reactions to children’s views”). Finally, questions were drafted using a daily needs terminology to identify the extent to which each obligation is met from the children’s perspectives. For example, the abovementioned construct was translated into the questions: “Do you feel free to share your opinions and feelings about the war/situation in public spaces?” “Have you experienced a physical assault by another person due to expressing your opinion in public spaces in the past 2 months?” (for a full description of the conceptualization of the various questions based on the CRC Articles and translated into daily needs of children, see Supplementary Table S1). As another example, Article 17, access to safe media, which is part of the participation rights cluster, creates an obligation to ensure delivery of child-appropriate information and to provide education and prevention regarding exposure to harmful content. Translating this to a day-to-day language of children’s needs, we asked: “To what extent has exposure to content and information caused you to feel distress or difficulty recently?” This question appeared following a list of various types of information sources, including social media.

The questionnaire also included sections addressing children’s well-being and demographic characteristics. We held a consultation session with a group of seven adolescent members of a Youth Advisory Board (YAB) in the Minerva Child and Youth Rights Program (ChYRP) at the Hebrew University, to ensure the questionnaire was accessible to adolescents. Further amendments were made to meet the ethical requirements of the Israeli Ministry of Education. An equivalent questionnaire was created for parents.

For the second wave of data collection, the questionnaire was translated into Arabic by a native Arabic speaker and a fluent Hebrew speaker (author HFZ). To enhance cultural and linguistic equivalence, the Arabic version of the questionnaire underwent a multi-step translation and consultation process. The Arabic translation was back-translated (Arabic to Hebrew), using two different AI language model platforms (OpenAI Chat GPT, GPT-4o; Claude Sonnet 4, version claude, *sonnet-4-20250514*). The first and last authors (both native Hebrew speakers) each compared one of the versions with the original Hebrew questionnaire and highlighted inconsistencies occurring in the translation process. Corrections were discussed among the research team until an agreement was reached. Following translation and back-translation, potentially ambiguous or culturally sensitive items—particularly those addressing fear, discrimination, exposure to violence, and the sociopolitical context—were reviewed in consultation with the Arabic-speaking Youth Advisory Board (YAB). Debatable items were shared with the ChYRP Arabic-speaking YAB, and their suggestions helped ensure readability and fidelity to the Hebrew version. YAB members identified unclear wording, suggested minor phrasing modifications, and provided concrete examples of how adolescents might interpret selected items. For several items, participants were also asked to translate them back into English and explain their meaning, enabling the research team to assess semantic clarity and emotional appropriateness. This process aimed to strengthen clarity, cultural relevance, and conceptual alignment with the original Hebrew version. Nevertheless, we acknowledge that, despite these efforts, subtle semantic differences reflecting culturally embedded meanings might persist and go undetected by all involved in this process, particularly when identical constructs are assessed across ethnic and language groups situated in different sociocultural, sociopolitical, and historical contexts.

Ultimately, the study employed Hebrew questionnaires for children and parents, and an Arabic children’s version, each included 60 items. Most items used a 0–7 scale to provide sufficient granularity for capturing nuanced experiences while remaining accessible to adolescents. Zero was included as an anchor point to allow respondents to indicate a complete absence of an experience (e.g., ‘not at all’). The 0–7 range provides eight response options, balancing discrimination capacity with cognitive load.

#### 2.2.2 Recruitment and distribution

Questionnaires were implemented on the Qualtrics platform for online distribution. A small-scale pilot study was conducted in a Jerusalem-based Hebrew-speaking school to ensure the readability of the questionnaire for adolescents and to verify that the technical procedure, including the pairing of child–parent dyads’ responses, was successfully implemented while maintaining participants’ anonymity. Although the pilot was successful and no amendments were needed, we subsequently excluded the data obtained in that phase due to the significant time gap with the start of data collection in Wave I.

The first wave of data collection took place from May 22nd to July 22nd, 2025, through a survey company. Parents received the questionnaires with an informed consent form, and were asked to forward a consent form and a questionnaire to their children. Participants were compensated with a voucher for a bookstore chain worth 30 NIS (~$9). This wave resulted in a sample that was predominantly Jewish (92%), Hebrew-speaking (89.9%), and consisted of only 4.5% participants from Arab minorities (Table 1), which was an insufficient representation and did not allow for meaningful group comparisons. We therefore aimed to collect another sample in Arabic.

In the second wave, we utilized both the Hebrew and Arabic versions to collect data from two additional samples (in Hebrew and Arabic) between August 1st and September 18th, 2025. The recruitment by the survey company yielded a sample of 101 responders to the Hebrew survey, but only a few responses to the Arabic version. Thus, we completed the recruitment of this group using a convenience sample and employed the snowball recruitment method. The convenience sampling approach used for the Arabic survey may introduce selection bias, limiting the generalizability of findings from this group. In addition, we received 40 responses, but only 24 participants eventually completed the questionnaire. Thus, the preliminary small-scale samples collected in Wave II were analyzed in an exploratory approach. Further work is in progress to complete an in-depth examination using the Arabic survey. Demographic characteristics of the Arabic sample (Table 1) indicate that participants were primarily Muslim (with one Christian participant). This sample composition does not capture the full spectrum of Arabic-speaking minorities in Israel, which include Druze, Bedouin, Christian, and other communities, limiting generalizability. This limitation is further discussed in the limitations section.

### 2.3 Measures

#### 2.3.1 Protection

Four item groups were used to measure the attainment of children’s rights to protection:

##### 2.3.1.1 Protection against violence

Participants were presented with a list of ways of physical/verbal violence and were asked to rate on a scale of 0–7 to what degree each harmed them in the past 2 months (0 = not at all, 7 = very much and very often), and the degree to which they received adequate support (0 = not at all, 7 = very much). They were also asked to detail whether there were places they feared or avoided.

##### 2.3.1.2 Best Interests

Participants were asked to rate on a scale of 0–7 the extent to which they felt that their various needs were fulfilled (0 = not at all, 7 = fully met), referring to physical safety, protection from harm by caretakers, sense of safety on social media, mental health support, health and safety for their family, access to information, education, and accommodation.

##### 2.3.1.3 Best interests during displacement

Participants were asked to rate (0–7) the extent to which they felt safe and had adequate physical conditions in their accommodation (0 = not at all, 7 = very much), and if there was any service that they were missing following displacement.

##### 2.3.1.4 Due process

Participants were asked if they had unwanted contact with the police, and if yes, to what degree they received fair treatment (0 = never/not at all fair; 7 = many times/very fair).

#### 2.3.2 Provision

Three item groups were used to measure the attainment of children’s rights to the provision of services:

##### 2.3.2.1 General needs

Participants were asked to rate to what extent they lacked basic necessities (e.g., food, clothing, housing, medical services; 0 = not at all, 7 = fully fulfilled), and to report which services they thought they should have received but did not.

##### 2.3.2.2 Education

Participants were asked: “Is your school currently operating?”; “Has the school staff changed since the outbreak of the war on October 7?” “Do you attend school regularly?”; “To what extent does the school understand and take your difficulties into consideration?”; “Does your school have adequate protective facilities?”

##### 2.3.2.3 Equality

Participants were presented with a list of services and were asked to rate the degree to which they felt that representatives of these services discriminated against them in the past 2 months (0 = not at all, 7 = very much). They were also asked to detail whether there was a reference to any characteristic of them in an instance of discrimination, and to what degree they tried to hide any such characteristics. Text comments were qualitatively analyzed.

#### 2.3.3 Participation and agency

Four item groups were used to measure the attainments of these rights:

##### 2.3.3.1 Privacy

Participants were presented with a list (disclosure of personal details, entry into private space, inspection of personal belongings, and intrusive security checks) and were asked to rate the degree to which they felt violations in each (0 = not at all, 7 = very much).

##### 2.3.3.2 Freedom of speech

Participants were asked to rate (0 = not at all, 7 = very much/very often) if they: feel free to express their opinions about the war in public, if they have experienced a physical or verbal assault due to expressing their opinion, harm to relationships with peers, or “shaming.” They were also invited to detail “If you do not feel free to share your opinions, what are you afraid of?,” and were offered to choose from a list of possible consequences: verbal or physical assaults, threats, shaming, boycott by peers, arrest, police violence, or “other.”

##### 2.3.3.3 Access to safe information

Participants were asked to rate information sources they use, and to report to what extent exposure to each has caused them distress (0 = not at all, 7 = very much).

##### 2.3.3.4 Participation in decisions regarding displacement

Participants were asked to report on the level of their involvement in decisions related to the new accommodation, school, and after-school activities (0 = I was not involved at all; 1 = I received information about the new situation; 2 = I expressed my opinion, but it was not taken into account; 3 = I was consulted, but the adults made the decision; 4 = The decision was made together by me and the adults; 5 = There was a joint discussion, and I made the decision; 6 = The decision was made by me alone; 7 = I initiated the decision, and the adults joined and supported).

#### 2.3.4 Wellbeing

Participants’ well-being was assessed using the KIDSCREEN-10 Index (Ravens-Sieberer et al., 2010), a 10-item questionnaire for children and adolescents. Both the child self-report version and the parent version assessing the child well-being were administered. Each item is rated on a 5-point Likert scale ranging from 1 (never/not at all) to 5 (always/extremely). Total scores are calculated by summing all items (range = 10–50; higher scores indicate better well-being). The KIDSCREEN-10 shows good reliability (Cronbach’s *α* = 0.82 for child self-report) and adequate test–retest reliability (ICC = 0.70) in international samples (Ravens-Sieberer et al., 2010). The Hebrew KIDSCREEN-10 has demonstrated internal consistency (Guttman’s Lambda ≥ 0.7) and good convergent validity (Barak et al., 2025).

### 2.4 Data analysis

All quantitative analyses were conducted using R version 4.4.0 (2024). Many continuous measures exhibited zero inflation (most participants reporting zero values). Zero-inflation is well-documented in sensitive topics research, where “excessive zeros in responses” create analytical challenges (Fulton et al., 2015). Given this distributional issue, our analytical approach differed depending on whether measures showed sufficient variability for statistical testing. For zero-inflated measures with limited variability, we report only the percentage of participants reporting any non-zero responses (prevalence of endorsement) as descriptive statistics, without conducting statistical comparisons between groups. For measures showing greater variability and more consistent distributions, we conducted statistical comparisons using permutation tests with false discovery rate (FDR) correction. Specifically, we used paired-sample permutation tests to assess differences between adolescents’ and parents’ reports in Wave I, and independent-sample permutation tests to compare groups in Wave II.

Missingness was also assessed to identify potential systematic patterns. For Wave I (parent–adolescent dyads), Chi-square tests revealed minimal associations with demographics (geography and gender) separately for parents and adolescents, supporting the missing-at-random assumption. Per-section missingness rates and zero percentages by group, and full test results, are presented in the Supplementary Tables. For Wave II (smaller independent adolescent samples, especially the Arabic survey), low power precluded such tests, but missingness rates and zero percentages by group (Hebrew vs. Arabic) are reported in the text and Supplementary Tables for each section. Missingness rates varied substantially across domains and groups. In Wave II, Arabic survey respondents showed consistently higher missingness rates across most domains compared to Hebrew survey respondents. The highest missingness rates were observed for sensitive topics such as violence exposure and discrimination experiences. Missingness rates by domain and group are reported in the text. Pairwise deletion was applied conservatively across both waves. Variables with ≥60% zeros (among all cases) were described by endorsement prevalence (% non-zero among respondents, unless stated otherwise). Variables with <60% zeros and reasonable non-zero distributions underwent permutation tests (10,000 iterations): paired tests for Wave I parent–adolescent comparisons (broman package; Broman, 2024) and unpaired tests for Wave II group or cross-wave comparisons (coin package; Hothorn et al., 2008). Cohen’s d and 95% permutation-based bootstrap confidence intervals are reported for all inferential results. The 60% threshold was empirically validated through distributional analysis (see Appendix A1), demonstrating that this threshold effectively separates variables with severe distributional violations (skewness >2.0, CV > 1.5) from those suitable for inferential testing. Full details and code are provided in the Supplementary materials.

Responses to open-ended items and text comments were analyzed in a qualitative approach. An inductive, data-driven approach was used to analyze the text responses provided by participants. A qualitative analysis was used to identify patterns in the data, which converged into key elements or categories (Birks and Mills, 2015). The analysis began with open coding, in which primary recurring patterns in the data were identified. Data extracts and initial categories suggested by the first author were audited by the last author, and disagreements were discussed. When categories were finalized, axial coding was formulated, and then a directed coding analysis continued until all the data were classified into categories. A theoretical integration was then made, and the finalized set of categories was grouped. For Wave I, saturation was evaluated by examining whether new categories emerged as additional responses were coded, continuing until no new categories emerged. For Wave II, particularly the Arabic survey with sparse responses, qualitative findings should be interpreted as preliminary patterns rather than saturated themes. Quotations were selected to represent the range of responses within each category, exemplifying core meanings while preserving participants’ original language.

As Wave II Arabic data rely on a very small convenience sample, both quantitative and qualitative analyses of cross-group comparisons in this wave are exploratory.

## 3 Results

Descriptive statistics are presented below, followed by inferential comparisons and qualitative findings where relevant.

### 3.1 Wave I

#### 3.1.1 Protection

##### 3.1.1.1 Protection against violence

**Figure 1:**
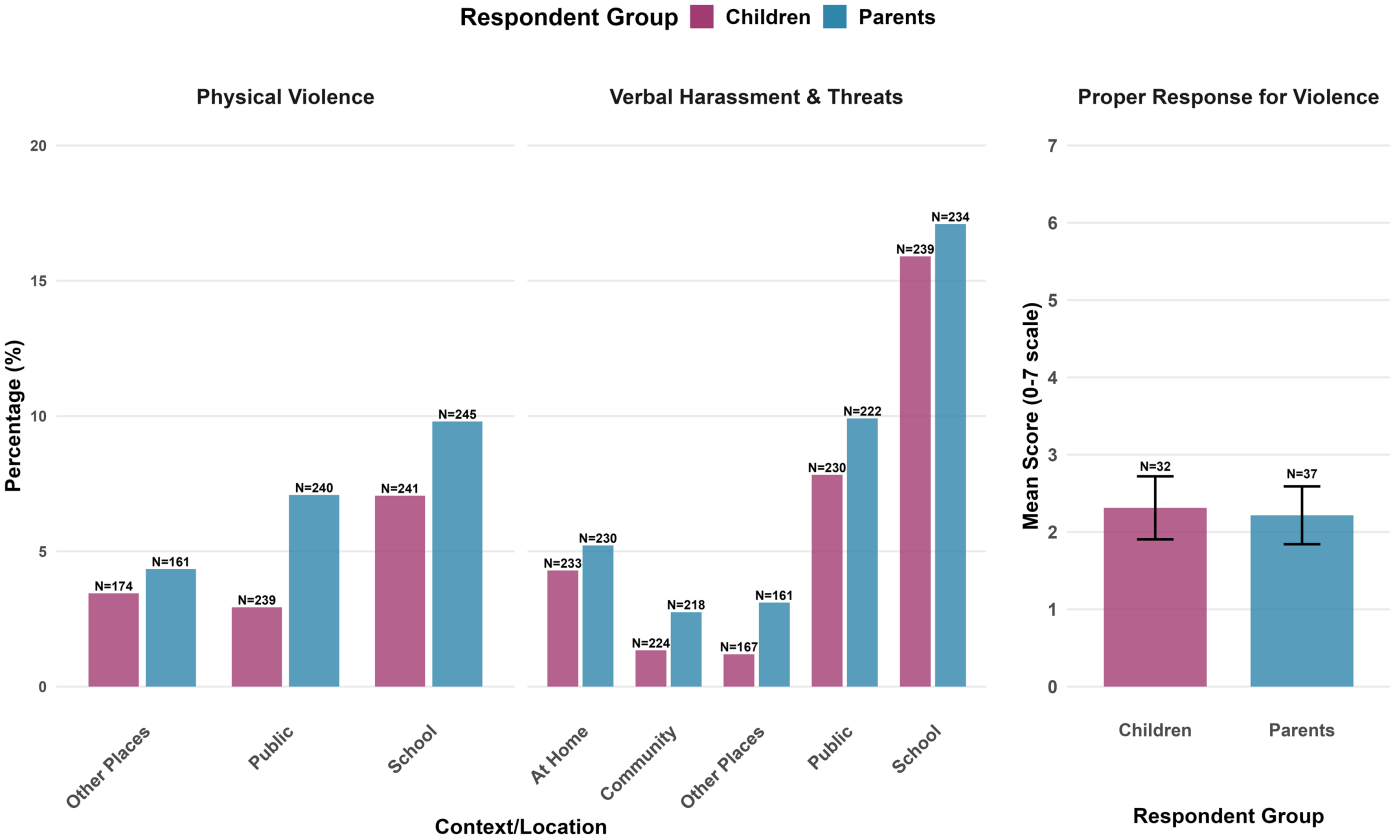
Violence exposure endorsement rates and institutional response satisfaction by context and respondent group. Error bars represent standard errors. Sample sizes are displayed above each bar. Percentages show endorsement rates among respondents (excluding missing data). Proper response scores reflect mean satisfaction (0–7 scale) among participants who reported violence.

Overall, 18.05% of the parents and 16.01% of the adolescents reported at least one instance of violence exposure across all contexts, with a high missingness rate in most questions (see Supplementary Table S1a for the full report). These measures were thus analyzed descriptively by reporting the percentage of endorsement (Figure 1). Overall, 20.1% of parents and 17.2% of adolescents reported at least one instance of violence exposure across all contexts. We present item-level results as percentages of respondents to each specific question (excluding missing data). Among those who answered, both adolescents and their parents most commonly reported adolescent exposure to verbal harassment/threats at school (parents: 17.09%, adolescents: 15.8%), with lower rates of physical violence reported across contexts. Among participants who reported violence exposure, satisfaction with institutional responses was generally low (Parents: *M* = 2.21, *SD* = 2.27, *N* = 37; Children: *M* = 2.31, *SD* = 2.30, *N* = 32).

When asked to rate place avoidance tendency due to fear, 46.4% of the adolescents reported no avoidance at all (0), 15.4% did not respond, and the rest (38.2%) reported varying levels of avoidance (*M* = 3.812, *SD* = 1.925). Ninety-nine adolescents detailed what they fear. A qualitative analysis revealed five categories: 1. Fear of the “other” [*N* = 53; fearing and avoiding Arab places and/or people (*n* = 41), travelling abroad (*n* = 8), fear of non-Jewish people (*n* = 2), of “anti-Semites” (*n* = 1), of “racist Jewish” people (*n* = 1)]; 2. Crowded places [*N* = 23; crowded places (*n* = 9), Tel Aviv (*n* = 7), public transportation (*n* = 3), shopping centers (*n* = 2), demonstrations (*n* = 2)]; 3. Isolated places and strangers [*N* = 19; dark places (*n* = 7), strangers and unknown places (*n* = 6), isolated places (*n* = 3), being alone among men (*n* = 2 female responders), “crime areas” (*n* = 1)]; 4. Proximity to war zone and conflict areas [*N* = 14; Judea and Samaria/The West Bank (*n* = 6), places with many rocket attack alerts (*n* = 4), areas near the borders (*n* = 3), war zone (*n* = 1)]. 5. Religious places and people [*N* = 13; Jerusalem/East Jerusalem/“the Old City” (*n* = 9), religious sites (*n* = 2), religious extremists (*n* = 2)]. Notably, the most prevalent sub-category was the fear of Arab “places” and people, with 41.4% of the responders mentioning fear of being in Arab towns and villages, and/or from Arab people. Two responses written in Arabic described fear of religious sites and “extremists.” The one response of fear from places with “racist Jewish” people was provided by a Muslim responder.

##### 3.1.1.2 Best interests

Participants reported varying levels of need fulfillment across domains, with accommodation receiving the highest rating (*M* = 6.42, *SD* = 1.39, *N* = 221), followed by family health and safety (*M* = 5.77, *SD* = 1.73, *N* = 255), physical safety (*M* = 5.61, *SD* = 1.69, *N* = 275), protection from harm by caretakers (*M* = 5.66, *SD* = 1.85, *N* = 177), information access (*M* = 5.39, *SD* = 1.76, *N* = 252), and educational needs (*M* = 5.27, *SD* = 1.80, *N* = 290). Lower fulfillment of needs was reported for social media safety (*M* = 4.92, *SD* = 1.87, *N* = 240). Mental health support needs showed the lowest response rate and lowest fulfillment ratings, although still above the theoretical midpoint of the scale (*M* = 4.08, *SD* = 2.47, *N* = 152).

##### 3.1.1.3 Best interests during displacement

Twenty-nine adolescents reported being evacuated from their homes after October 7th 2023. Of them, 23 were already back home at the time of the survey completion. In rating how safe they feel and whether there were adequate conditions where they live on a scale of 0–7, the mean score above the theoretical midpoint for both feeling safe (*M* = 5.41, *SD* = 2.18, *N* = 294) and having adequate physical conditions (*M* = 5.31, *SD* = 2.21, *N* = 29). Parents’ reports were similar (safety: *M* = 5.08, *SD* = 1.57, *N = 26*; conditions: *M* = 5.85, *SD* = 1.46, *N = 26*). No significant differences were found between the reports of adolescents and their parents (*p* = 356, 95% CI[−1.26,0.462]; *p* = 0.359, 95% CI[−0.34,1.38]). Few adolescents responded to the question about whether there was any service they lack (*n* = 5). Of them, four mentioned after-school activities, and one mentioned learning and social activities.

##### 3.1.1.4 Due process

Thirteen respondents reported having unwanted contact with the police. Of them, four reported receiving unfair treatment. Detailed descriptions included contact with the police after an accident, and contact due to a wanted suspect contacting the child. Interestingly, only six parents reported their child to have experienced unwanted contact with the police; of them, five reported that their child’s right for a fair process was not at all or hardly harmed (0–3), and only one parent reported an unfair process. This anecdotal observation implies that parents’ and children’s reports might not align. However, we were unable to perform statistical analysis for such small sample sizes.

#### 3.1.2 Provision

##### 3.1.2.1 General needs

When asked to rate the extent to which their family lacks basic needs, 60.5% of the adolescents reported no deprivation at all (0), 14.7% did not respond (missing data), and the rest reported varying levels of deprivation ranging from 1–7 (*M* = 3.68, *SD* = 2.09, *N* = 76). Seventy-six adolescents provided text responses, which fall under eight categories: 1. School and learning (*n* = 19), e.g., “help with my studies”; 2. Afterschool activities (*n* = 16), e.g., “afterschool activities that suit my age”; 3. Leisure time and social activities (*n* = 14), e.g., “friends,” “happy events”; 4. Emotional support (*n* = 13), e.g., “a psychologist in school”, “mental help”; 5. Financial support and job opportunities (*n* = 7), e.g.: “We should have been compensated for living away from home and having to live in bad conditions”, “more job opportunities”; 6. Access to information (*n* = 6), e.g., “clear information”, “more lessons in school about the situation”; 7. Medical services (*n* = 4), e.g., “available appointments for clinics”; 8. Six adolescents mentioned the service they need is to “talk to someone”, and “have conversations with someone who understands my needs”.

##### 3.1.2.2 Education

According to adolescents’ reports, most schools (99.7%) were operating while they completed the questionnaire. Adequate protective facilities concerns were reported by 21.6% of children and 27.9% of parents. Staff changes affected 23.3% participants, with textual responses revealing widespread teacher departures due to military reserve duty. School attendance irregularities were reported by 16% of adolescents and 12.2% of parents. Children reported moderate levels of perceived school understanding and considering their difficulties (*M* = 4.27, *SD* = 2.07, *N* = 299), slightly above the theoretical midpoint of the used scale.

##### 3.1.2.3 Equality

**Figure 2:**
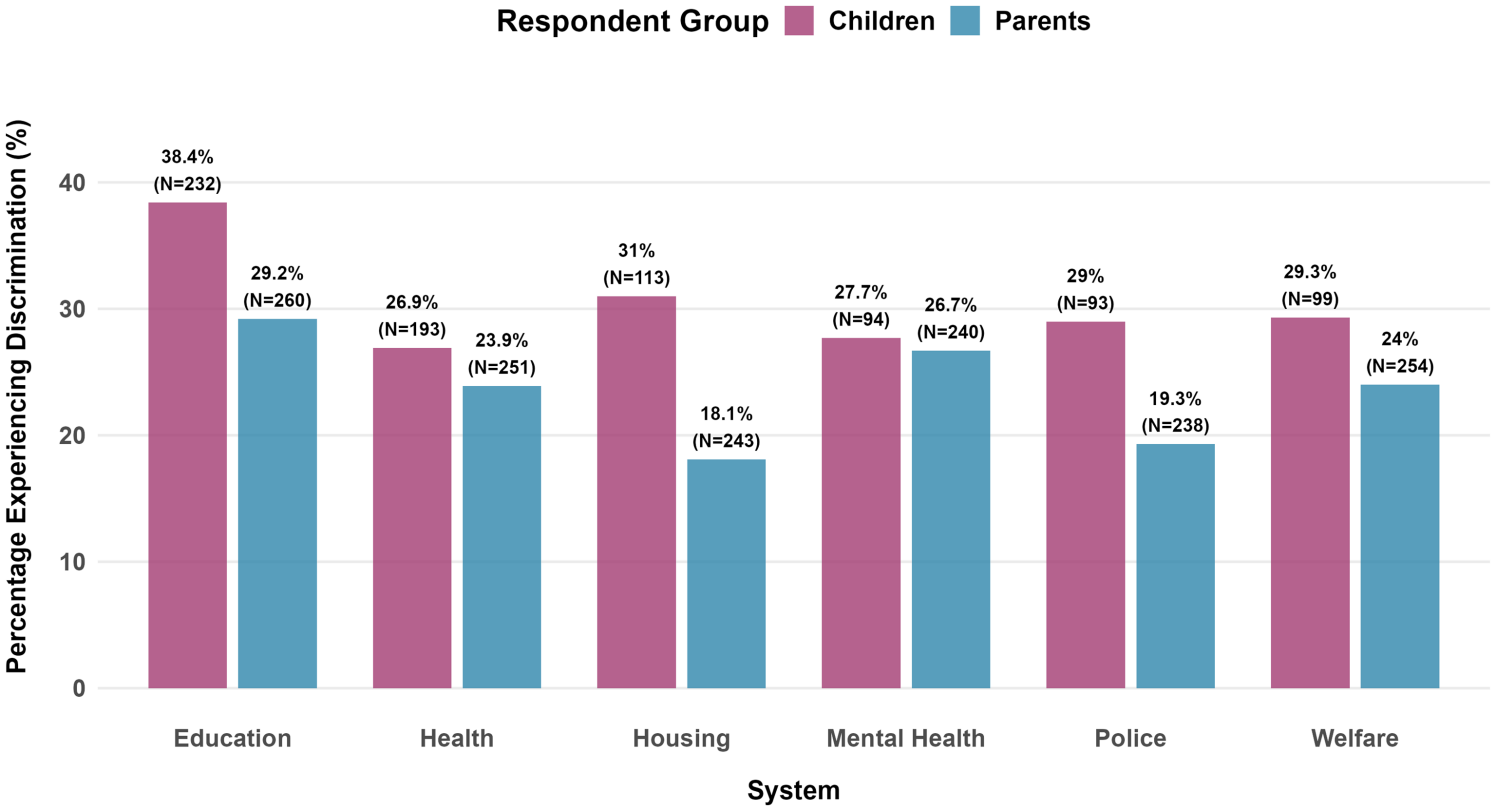
Discrimination experience by respondent group. Sample sizes reflect the number of participants who provided responses to each question. Percentages represent the proportion of respondents reporting any discrimination experience within each institutional system.

Overall, 33% of parents and children reported at least one non-zero discrimination experience. Given the predominance of zero responses, percentages of non-zero responses are presented as the outcome measure. Figure 2 presents comprehensive endorsement rates and response patterns. Adolescents showed higher missingness rates (24.2–69.3%) compared to parents (15–23%), with the largest gap in mental health discrimination (69.3% vs. 23% missing). This differential missingness pattern should be taken into account when interpreting group comparisons. Among participants who did respond, children typically reported higher discrimination rates than parents in education (38.4% vs. 29.2%), housing (31.3% vs. 18.1%), and police (29% vs. 19.3%) - with mental health being an exception: parents reported slightly more discrimination (27.7% vs. 26.7%).

When asked to detail whether there was a reference to any characteristic of them on the occasion of discrimination, 48 adolescents provided details. Of them, most responders (n = 20) reported that their physical appearance was referred to, with reports including: being “pretty,” “colored,” and “overweight.” Eighteen responders reported that gender was referred to when being discriminated against (six girls, three boys, and the rest did not state their gender). Seventeen responders reported reference to nationality. Five specified that the discrimination was based on them “being Jewish,” three on “being Israeli,” and two on “being Arab.” Notably, adolescents mentioned discrimination on social media when relating to nationality. Fifteen responders mentioned religion (being “Jewish,” “being secular/non-religious,” “being Muslim”). Fourteen responders noted that their ethnic origin was mentioned, including being “Ashkenazi,” “Mizrachi,” Russian, English, Arab, and Bedouin. Fifteen participants noted their age, with one responder detailing, “I am not being taken seriously because of my age.”

Adolescents were further asked if they conceal characteristics that might lead to discrimination. Many (15.08%) did not respond to this question, and 66% answered that they do not at all try to do that (0). Among the 18.9% who reported conceal characteristics, responses ranged from 1 to 7 (very much), with an overall low mean score (*M* = 2.87, *SD* = 1.97), indicating negligible reported engagement in this behavior among adolescents in our sample.

#### 3.1.3 Participation and agency

##### 3.1.3.1 Privacy

Overall, 25.1% of parents and 19.9% of adolescents reported experiencing at least one privacy violation of the adolescents across all measured items. Across the four privacy-violation types, children consistently had higher rates of missing data compared to parents. For disclosure of personal information, 41.5% of children had missing data compared to 31.4% of parents, with children reporting lower endorsement rates among those who responded (12.3% vs. 28.6%). Invasion of private space showed similar missing data rates for both groups (Parents: 33.3%, Children: 34.3%) and nearly identical endorsement rates (adolescents: 24.4%, parents: 24%). For examination of private materials, children again demonstrated higher missing data rates (39.5% vs. 37.3%) and lower endorsement rates (14.1% vs. 21.9%). Intrusive security checks exhibited the highest missingness for both parents (43.5%) and adolescents (44.1%), with a notable difference in endorsement rates among respondents (adolescents: 8.2%, parents: 18.5%).

##### 3.1.3.2 Freedom of speech

**Figure 3:**
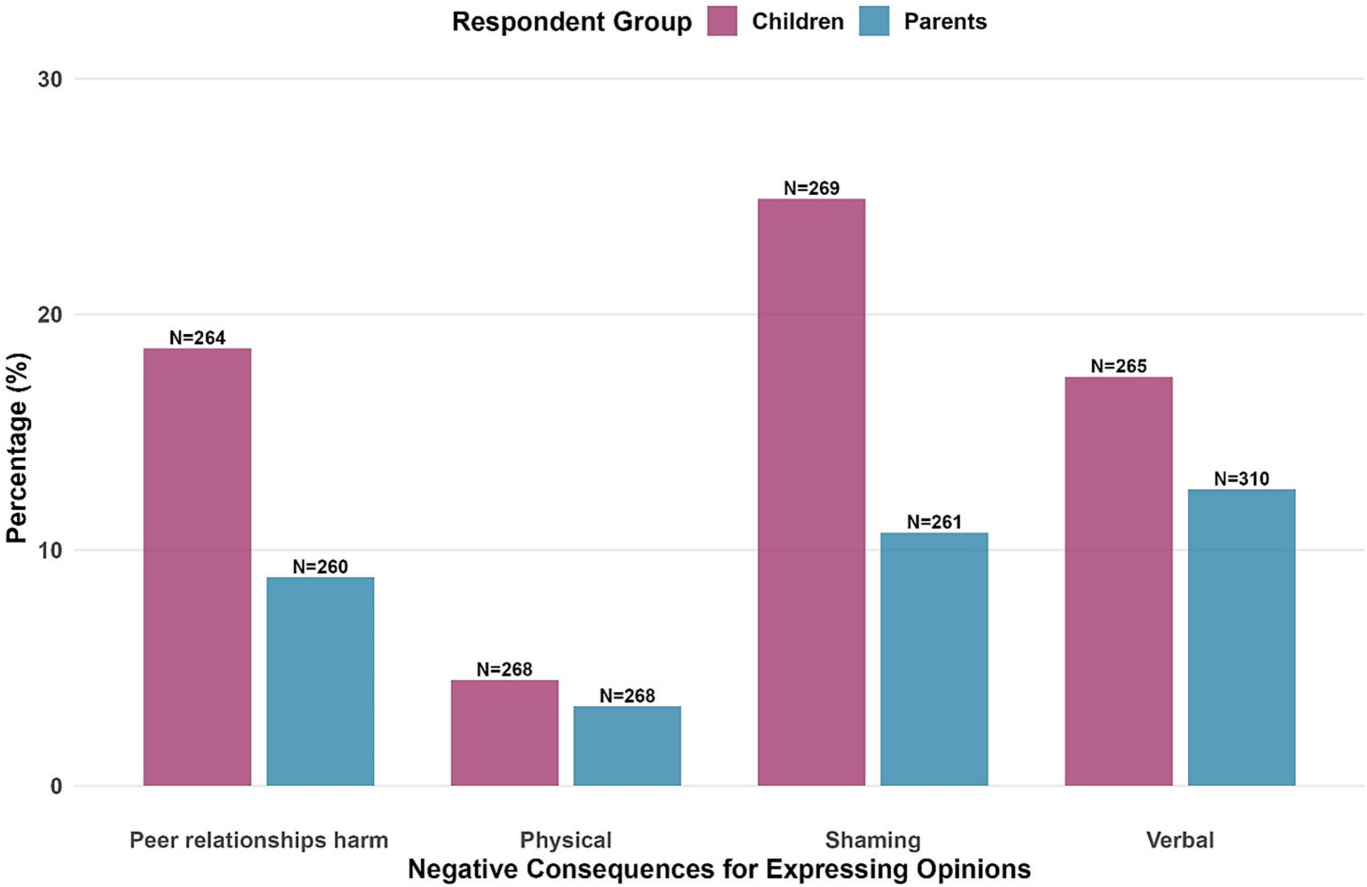
Negative consequences experienced for expressing opinions about the war. Sample sizes reflect the number of participants who responded to each question. Percentages represent the proportion of respondents reporting any assault in response to sharing their opinions.

Parents reported their children as freer to share their opinions about the war (*M* = 5.05, *SD* = 1.914) compared to adolescents’ own self-reports (*M* = 4.43, *SD* = 2.40). This difference was significant using a paired permutation test (95% CI [0.34,0.91], *d* = 0.25, *p* < 0.001). Given zero inflation in responses to the question of whether adolescents experienced negative consequences due to expressing their opinion, we report percentages of endorsement rather than means (Figure 3). Overall, 26.1% of children and 17.3% of parents reported that the adolescents experienced any of such consequences. Adolescents reported experiencing shaming as the most common consequence (23.1% of respondents), followed by peer relationship harm (18.4%) and verbal assault (17.24%). Physical assault was rarely reported (5.1%). Parents consistently underestimated their children’s experiences, reporting lower rates for shaming (9.5%), peer relationship harm (9.6%), verbal assault (11.4%), and physical assault (3.4%).

More than half of the adolescents did not respond to the question “If you do not feel free to share your opinions, what are you afraid of?” of the 127 who did respond, 46% mentioned verbal assaults, 37% mentioned fear of being boycotted by peers, 34% fear of shaming, 27% of physical assault, 22% of threats, 7% police violence, and 6% mentioned the fear of arrest. “Other” reasons included fear of “not being accepted,” “not being loved,” “having arguments,” “being hurt,” and not wanting to express an opinion due to “not being knowledgeable enough.”

##### 3.1.3.3 Access to safe information

**Figure 4:**
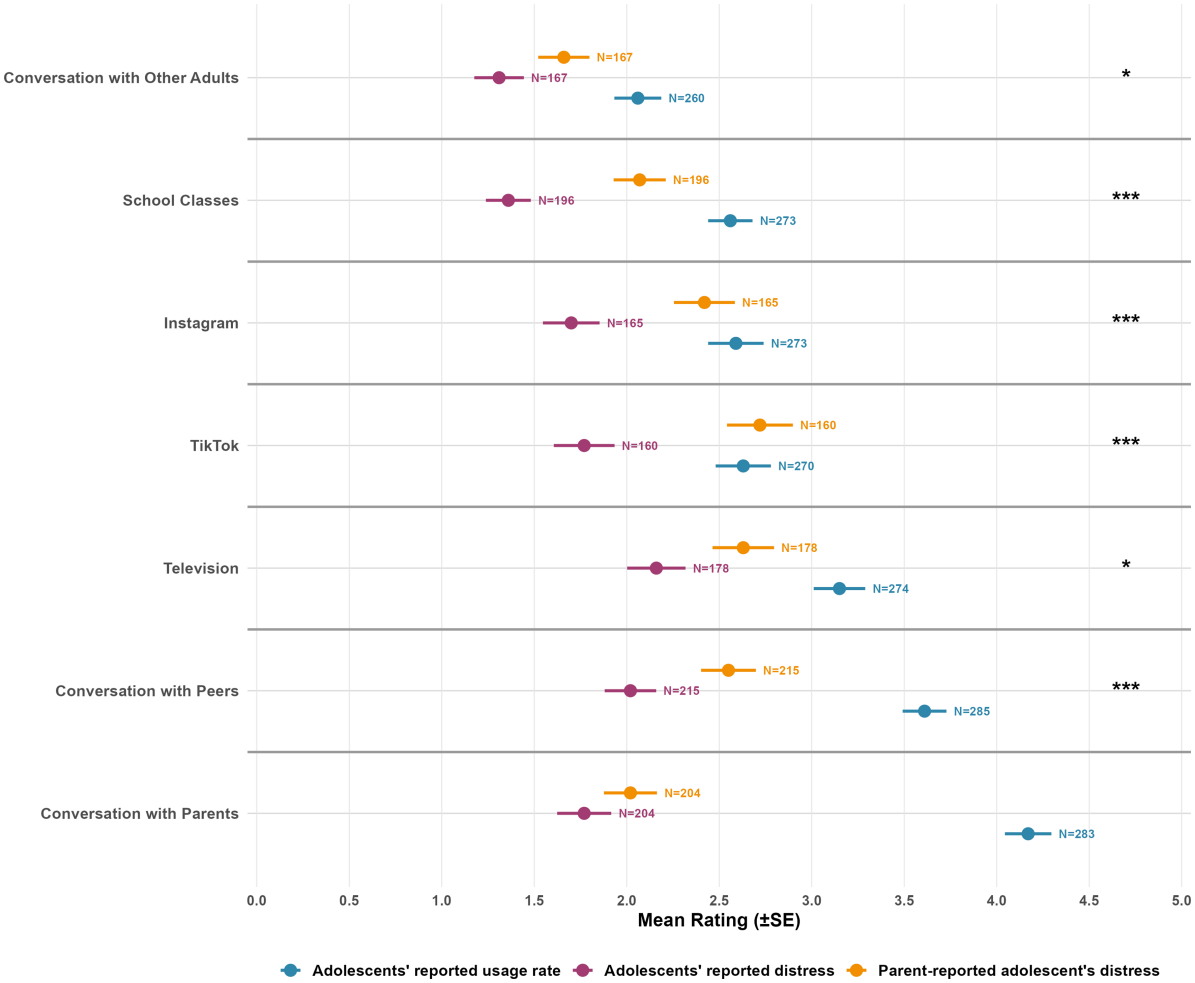
Information source usage frequency and associated distress levels among children. Error bars represent standard error. *N* = indicates the number of valid responses for each measure. Usage frequency (blue) and distress levels (purple for children, yellow for parents) are shown for 13 information sources. Distress measures consistently showed higher missingness rates (26–65%) compared to usage measures (7–27%), indicating differential response willingness. Sources are ordered by usage frequency from highest to lowest. * = significant after FDR correction.

Adolescents reported that conversations with parents were the most frequently used source of information about the war (*M* = 4.17, *SD* = 2.11, *N* = 283), followed by conversations with peers (*M* = 3.61, *SD* = 2.00, *N =* 285) and television (*M* = 3.15, *SD =* 2.31, *N = 274*) and TikTok and Instagram were reported to be less used (*M* = 2.63, *SD* = 2.45, *N* = 270 and *M* = 2.59, *SD* = 2.48, *N* = 273, respectively), as well as school classes (*M* = 2.5.6, *SD =* 1.98*, N =* 273) and conversations with other adults (*M* = 2.06, *SD =* 2.04*, N =* 260). Other sources were reported to be hardly used (Telegram, Facebook, X, and online and printed newspapers). Next, we wanted to compare the distress each information source evoked for adolescents as reported by themselves versus their parents. In line with the consistent strategy across this paper, we included only sources which at least 60% of adolescents reported using (non-zero usage), and for which both distress measures (by both parents and children) showed more than 60% non-zero responses (see Supplementary Table S4 for full results). Seven sources met these criteria: Instagram, TikTok, television, conversation with parents, conversation with peers, school classes, and conversation with other adults (see Figure 4). While conversations with parents and peers were reported to be most used for information about the war, they were not reported to cause more distress than other sources; thus, the gap between information consuming and feeling of distress was most notable for these interpersonal sources (Figure 4).

Parents systematically assessed sources of information as more distress-evoking than what adolescents reported experiencing. Paired permutation tests revealed significant differences for 6 of 7 sources after FDR correction: TikTok (*M*_Parents_ = 2.72, *SD* = 2.25 vs. *M*_Adolescents_ = 1.77, *SD* = 2.08; *n* = 160 pairs, *d* = 0.42, 95% CI [0.61, 1.31], *p* < 0.001), school classes (*M*_Parents_ = 2.07, *SD* = 1.97 vs. *M*_Adolescents_ = 1.36, *SD* = 1.70; *n* = 196 pairs, *d* = 0.33, 95% CI [0.42, 1.01], *p* < 0.001), Instagram (*M*_Parents_ = 2.42, *SD* = 2.12 vs. *M*_Adolescents_ = 1.70, *SD* = 1.97; *n* = 165 pairs, *d* = 0.35, 95% CI [0.41, 1.04], *p* < 0.001), conversation with peers (*M*_Parents_ = 2.55, *SD* = 2.17 vs. *M*_Adolescents_ = 2.02, *SD* = 2.04; *n* = 215 pairs, *d* = 0.23, 95% CI [0.22, 0.85], *p* < 0.001), television (*M*_Parents_ = 2.63, *SD* = 2.22 vs. *M*_Adolescents_ = 2.16, *SD* = 2.10; *n* = 178 pairs, *d* = 0.20, 95% CI [0.12, 0.80], *p* = 0.011), and conversation with other adults (*M*_Parents_ = 1.66, *SD* = 1.79 vs. *M*_Adolescents_ = 1.31, *SD* = 1.73; *n* = 167 pairs, *d* = 0.21, 95% CI [0.10, 0.60], *p* = 0.011). Conversation with parents showed no significant difference (*M*_Parents_ = 2.02, *SD* = 2.05 vs. *M*_Adolescents_ = 1.77, *SD* = 2.09; *n* = 204 pairs, *d* = 0.11, 95% CI [−0.07, 0.56], *p* = 0.123).

##### 3.1.3.4 Participation in decisions regarding displacement

In rating the degree to which children participated in decision-making regarding their displacement answers ranged 0–7 (“I was not involved at all”–“I initiated the decision, and the adults joined and supported”), with a mean score slightly above the theoretical mid-point for all three measures: “where will be our new accommodation” (*M* = 3.82, *SD* = 2.36, *N* = 22), “what will be my new school” (*M* = 4.39, *SD* = 2.33), and “what will be my new after-school activities” (*M* = 4.47, *SD* = 2.45). When asked about the level of participation of their child in decision-making regarding displacement, parents’ reports yield similar mean scores: new accommodation: *M* = 3.47, *SD* = 2.31 (slightly below the theoretical midpoint); new school: *M* = 4.17, *SD* = 2.26, *N* = 18; and after-school activities: *M* = 3.47, *SD* = 2.17. Inferential statistics were not applicable (*n* = 29 in each reporter’s group, 25–47% missing data across measures). Importantly, most families had already returned home by the time the survey was completed (74%). However, an interesting qualitative observation is that only 2 adolescent and only 2 parent reported zero participation of the adolescent in decision making, while full participation was reported 13 times by adolescents and nine times by parents. This implies that, in the current small sample of displaced adolescents, those who chose to report their involvement in decision-making reported participation, and their parents’ reports converged with this pattern.

#### 3.1.4 Wellbeing

Reports from parents and adolescents on the adolescents’ well-being were similar (*M*_Adolescents_ = 25.71, *SD* = 4.40, *N* = 296; *M*_Parents_ = 25.87, *SD* = 4.04). Participants with missing items were excluded from the analysis. A paired permutation test (Broman, 2024) comparing the mean scores revealed no significant difference between adolescents’ own reports and parental reports (*p* = 0.45, 95% CI [−0.26,0.58]). Parent assessments of their child’s well-being were also strongly and positively correlated with children’s self-reports (*r* = 0.62, *p* < 0.001). The sample means for both adolescents’ and parental reports fall below the theoretical midpoint (30) of the scale used to assess well-being levels.

### 3.2 Wave II – exploratory analyses

Missingness patterns in Wave II: Arabic survey respondents demonstrated higher missingness rates across most domains compared to Hebrew survey respondents. The highest missingness was observed for violence-related items (Arabic: 41.7–83.3%, Hebrew: 25.7–60.4%), followed by discrimination experiences (Arabic: 25–42%, Hebrew: 25–31%). Missingness rates for each domain are reported in the relevant sections below.

#### 3.2.1 Protection

##### 3.2.1.1 Protection against violence

**Figure 5:**
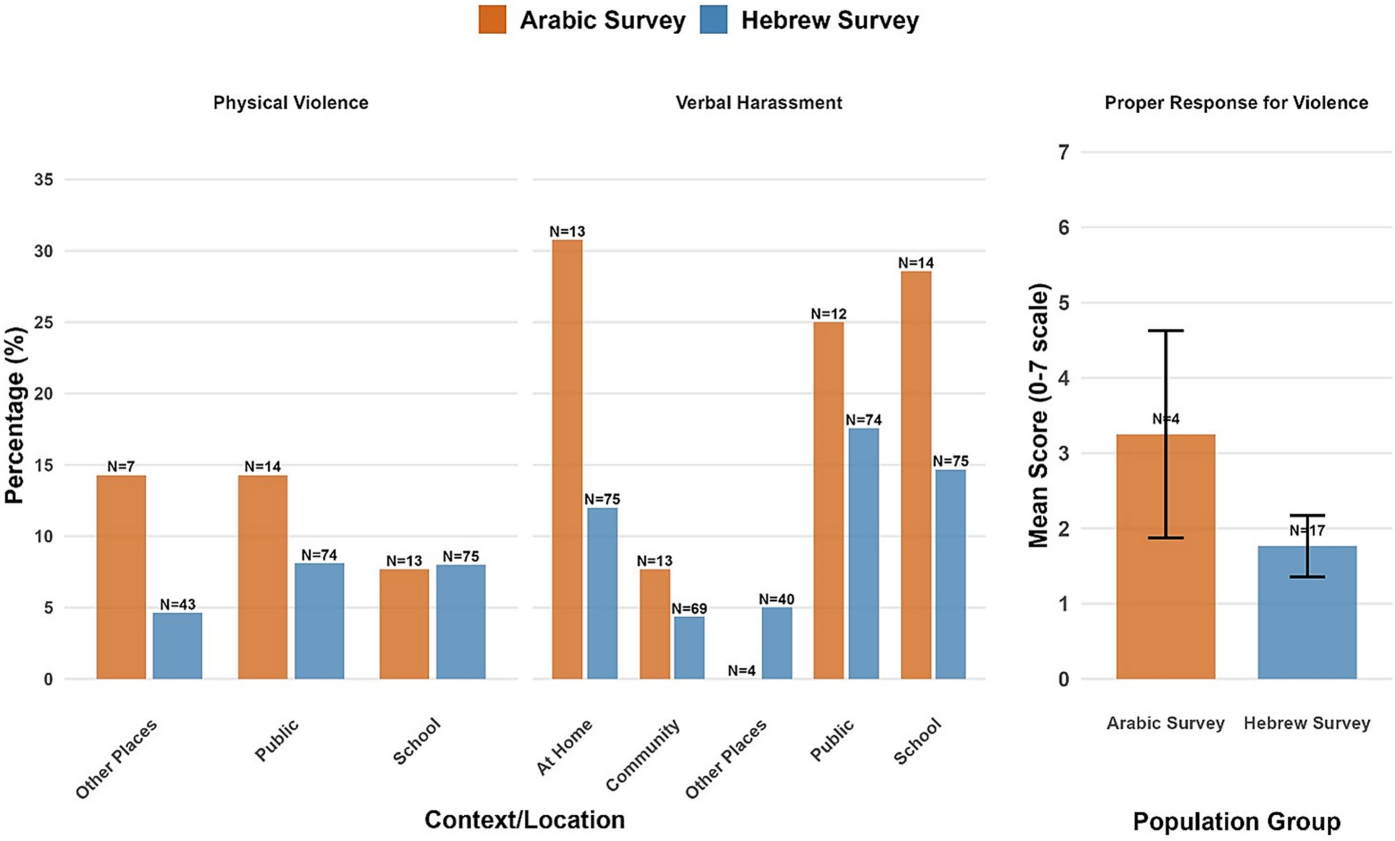
Violence exposure and institutional response by population group and context. Error bars represent standard errors. Sample sizes are displayed above each bar. Percentages show non-zero endorsement rates among respondents who answered each question (excluding missing data). Proper response for violence scores reflects mean satisfaction ratings (0–7 scale) among participants who reported any violence exposure and were eligible for the institutional response question.

Overall, 25.0% of respondents to the Arabic survey and 25.7% of the Hebrew survey reported at least one non-zero exposure to violence across all measured contexts. Figure 5 presents comprehensive endorsement rates across all variables by group, showing response rates for each question. Responders to the Arabic survey demonstrated higher non-response rates across contexts (41.7–83.3% missing) compared to responders to the Hebrew survey (25.7–60.4% missing). In this exploratory sample, among responders to the Arabic survey, higher percentages of verbal harassment were reported, particularly at home (32% of respondents), compared to the Hebrew survey. However, these estimates are based on very small samples (*N* = 4–13 per question in the Arabic survey, *N = 32–75* per question in the Hebrew survey). Among participants who reported exposure to violence (Arabic: *N* = 6, Hebrew: *N* = 26), satisfaction with institutional responses was lower in the Hebrew sample than in the Arabic sample (Arabic: *M* = 4.33, *SD* = 2.08, *N* = 3; Hebrew: *M* = 2.73, *SD* = 1.20, *N* = 11), although the very small sample size limits the interpretability of these findings.

*Avoiding places:* When asked about attempts to avoid places due to fear, most participants reported zero avoidance or did not respond. Given these zero inflation rates, we report percentages of endorsement and descriptive statistics for those who reported avoidance. Overall, 37.5% of Arabic survey respondents and 33.7% of Hebrew survey respondents reported avoiding some places at least to some degree (1–7) (calculated from the entire sample, including non-responders). High missingness rates were observed (Arabic: 37.5%, Hebrew: 16.8%).

In the Hebrew sample, twenty-two adolescents provided details. Responses fall into similar categories as in Wave I: 1. Fear of the “other” was the largest category [*N* = 14; fearing and avoiding Arab places or people (*n* = 11), fear of travelling abroad (*n* = 2), fear of “anti-Semites” (*n* = 1)]; 2. Crowded places [*N* = 2]; 3. Proximity to the war zone and areas of conflict [*N* = 4; Judea and Samaria/The West Bank (*n* = 2), areas near the borders (*n* = 3), war zone (*n* = 2)]; 4. Religious places and people [*N* = 2; Jerusalem/East Jerusalem/“the Old City” (*n* = 2)]. One respondent also mentioned “school” as a place they avoid, and another respondent noted avoiding “sad places.” Among the responders to the Arabic survey, only four provided details. Their answers included “Dangerous places or places where I feel unwelcome, such as places that are different from me in language, origin, or religion”, and: “Fear of racism or acts of violence as a result of the current situation and social tension”, which fall under the category of *fear of the “other*.”

##### 3.2.1.2 Best interests

Comparison of the level of need fulfillment across domains between Wave I (adolescents’ self-reports) and Wave II (combined Arabic and Hebrew) revealed one significant difference after FDR correction. Wave II respondents reported lower education-related needs fulfilment (*M*_Wave II_ = 4.63, *SD* = 2.20, *n* = 115) compared to Wave I (*M*_Wave I_ = 5.27, *SD* = 1.80, *n* = 290; difference = 0.64, 95% CI [0.19, 1.09], *d* = 0.33, *p* = 0.018), and lower social media safety needs fulfillment compared to Wave I (*M*_Wave II_ = 4.22, *SD* = 2.08, *n* = 90; *M*_Wave I_ = 4.92, *SD* = 1.87, *n* = 240; difference = 0.70, 95% CI [0.21, 1.18], *d* = 0.36, *p* = 0.018) Other needs showed similar patterns [physical safety (*M*_Wave II_ = 5.43, *SD* = 2.11, *n* = 101; *M*_Wave I_ = 5.61, *SD* = 1.69, *n* = 275; difference = 0.18, *p* = 0.435), mental health (*M*_Wave II_ = 3.42, *SD* = 2.46, *n* = 67; *M*_Wave I_ = 4.08, *SD* = 2.47, *n* = 152; difference = 0.66, *p* = 0.135), health (*M*_Wave II_ = 5.52, *SD* = 1.96, *n* = 91; *M*_Wave I_ = 5.79, *SD* = 1.75, *n* = 244; difference = 0.27, *p* = 0.306), family health (*M*_Wave II_ = 5.85, *SD* = 1.72, *n* = 92; *M*_Wave I_ = 5.77, *SD* = 1.73, *n* = 255; difference = −0.08, *p* = 0.722), information access (*M*_Wave II_ = 4.94, *SD* = 2.04, *n* = 93; *M*_Wave I_ = 5.39, *SD* = 1.76, *n* = 252; difference = 0.45, *p* = 0.135), protection from harm by caretakers (*M*_Wave II_ = 5.11, *SD* = 2.56, *n* = 71; *M*_Wave I_ = 5.66, *SD* = 1.85, *n* = 177; difference = 0.55, *p* = 0.135), and permanent accommodation (*M*_Wave II_ = 6.08, *SD* = 1.89, *n* = 79; *M*_Wave I_ = 6.42, *SD* = 1.39, *n* = 221; difference = 0.34, *p* = 0.148)] with no significant differences after correction for multiple comparisons.

**Figure 6:**
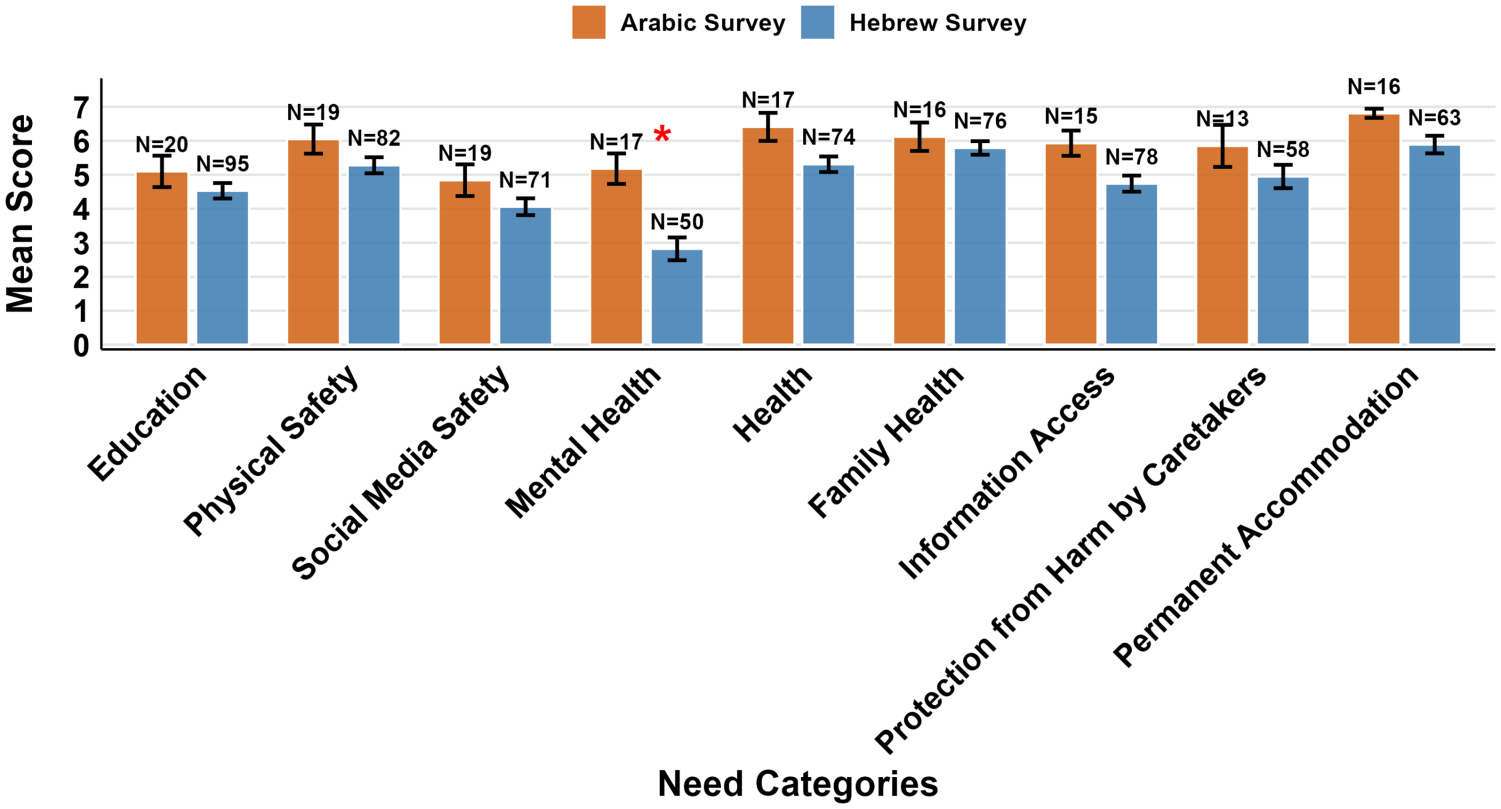
Mean ratings of nine need categories by group. Error bars represent standard error. *N* = number of respondents. *p < 0.05 after FDR correction for multiple comparisons.

Groups in wave II were compared using permutation tests across categories. Only mental health needs showed a statistically significant difference after FDR correction for multiple comparisons (*Z* = 3.408, *p* = 0.006), with respondents to the Arabic survey reporting higher fulfillment of mental health needs (*M*_Arabic_ = 5.18, *SD* = 1.85, *N* = 17, Zero% = 5.9%) compared to those in the Hebrew survey (*M*_Hebrew_ = 2.82, *SD* = 2.37, *N* = 50, Zero% = 24%; difference = 2.36, 95% CI [1.23, 3.38], *d* = 1.05). Other needs showed non-significant patterns: education (*M*_Arabic_ = 5.10, *SD* = 2.07, *N* = 20; *M*_Hebrew_ = 4.53, *SD* = 2.22, *N* = 95; *p* = 0.360), physical safety (*M*_Arabic_ = 6.05, *SD* = 1.87, *N* = 19; *M*_Hebrew_ = 5.28, *SD* = 2.14, *N* = 82; *p* = 0.248), social media safety (*M*_Arabic_ = 4.84, *SD* = 2.03, *N* = 19; *M*_Hebrew_ = 4.06, *SD* = 2.08, *N* = 71; p = 0.248), health (*M*_Arabic_ = 6.41, *SD* = 1.70, *N* = 17; *M*_Hebrew_ = 5.31, *SD* = 1.97, *N* = 74; *p* = 0.134), family health (*M*_Arabic_ = 6.12, *SD* = 1.67, *N* = 16; *M*_Hebrew_ = 5.79, *SD* = 1.74, *N* = 76; *p* = 0.536), information access (*M*_Arabic_ = 5.93, *SD* = 1.44, *N* = 15; *M*_Hebrew_ = 4.74, *SD* = 2.09, *N* = 78; *p* = 0.134), protection from harm by caretakers (*M*_Arabic_ = 5.85, *SD* = 2.23, *N* = 13; *M*_Hebrew_ = 4.95, *SD* = 2.62, *N* = 58; *p* = 0.336), and permanent accommodation (*M*_Arabic_ = 6.81, *SD* = 0.54, *N* = 16; *M*_Hebrew_ = 5.89, *SD* = 2.06, *N* = 63; *p* = 0.198). However, given the substantial sample size disparity (*N*_Arabic_ = 13–20 vs. *N*_Hebrew_ = 50–95), findings should be considered exploratory (Figure 6).

##### 3.2.1.3 Best interests during displacement

From the responders to the Hebrew Survey, four reported being displaced. Of them, two were already back home at the time of the survey completion. In rating safety and adequate physical conditions, one participant reported a level of 2 (on a scale of 0–7; for both questions), two reported the highest level of safety and adequate conditions (7), and one reported the highest level of adequate conditions (7), with a somewhat lower sense of safety (5). Three reported that nothing was missing, and one mentioned the need for age-appropriate activities. No respondent to the Arabic survey reported being evacuated.

##### 3.2.1.4 Due process

In the Hebrew survey, four respondents reported having unwanted contact with the police. Of them, two reported receiving unfair treatment (levels 1–3 of a 0–7 scale). No respondent provided any details. In the Arabic survey, three respondents reported having unwanted contact with the police. Of them, two reported receiving unfair treatment (levels 2–3). One respondent detailed that the contact was due to harm caused by their parents. The very small numbers did not allow for any analyses. However, the anecdotal observation of four responses out of 101 participants (3.9%) in the Hebrew survey, compared with three responses out of 24 participants (12.5%) in the Arabic survey, warrants further research.

#### 3.2.2 Provision

##### 3.2.2.1 General needs

Most participants reported zero deprivation or did not respond. The endorsement and descriptive statistics for those who reported any deprivation indicate that overall, 25% of Arabic and 21.8% of Hebrew survey respondents reported at least some basic needs deprivation (calculated from the entire sample, including non-responders). High missingness rates were observed (Arabic: 20.8%, Hebrew: 24.8%). Among those who reported any basic needs deprivation, Hebrew survey respondents showed a higher intensity of deprivation (*M* = 3.14, *SD* = 1.88, *N* = 22) compared to Arabic survey respondents (*M* = 2.83, *SD* = 2.56, *N* = 6).

Thirty-nine of the respondents to the Hebrew survey provided details on what they need. Of them, 22 noted that they believe they received all necessary services, or that they “do not know.” The remaining 17 responses fall under the following categories: 1. School and learning (*n* = 1; “one-on-one tutoring from school”); 2. Afterschool activities (*n* = 6, e.g., “sport,” “arts”); 3. Leisure time and social activities (*n* = 4; “having fun,” “other youth”); 4. Emotional support (*n* = 3; “professional help,” “psychologist who can help with stress and emotional eating”); 5. One adolescent mentioned the need to “be talked to more openly in school.” Eight of the respondents to the Arabic survey responded to this item. Of them, three noted that they believe they received all necessary services. The five remaining responses included “a math teacher” (School and learning), “trips” (Leisure time and social activities), “some clothes and shoes” (Financial support), and two adolescents mentioned that they needed more “safety” and “less fear from the future.”

##### 3.2.2.2 Education

Respondents in both groups reported similar shelter adequacy and protection in school facilities (Arabic: 70% reported adequate protection, Hebrew: 71.7%). In this exploratory sample, respondents to the Arabic survey reported lower rates of teaching staff changes (10% vs. 27% for respondents to the Hebrew survey), higher ratings by respondents to the Arabic survey were also reported for school support (Arabic: *M* = 4.25, *SD* = 2.36, *N* = 20; Hebrew: *M* = 3.75, *SD* = 2.20, *N* = 99). A permutation test indicated a non-significant difference between groups (*Z* = 0.92, *p* = 0.378, 95% CI [−0.57, 1.61]).

##### 3.2.2.3 Equality

**Figure 7:**
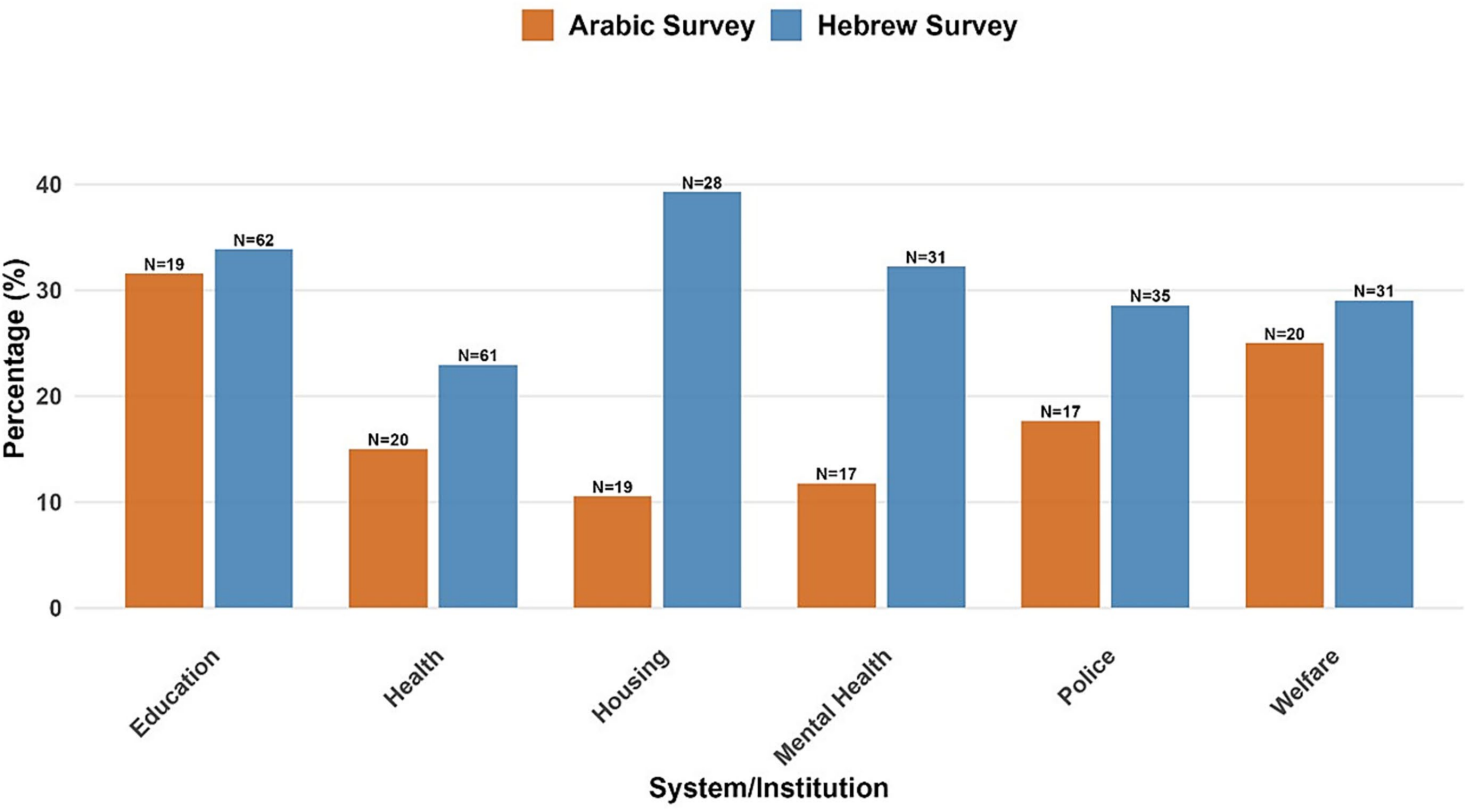
Discrimination experience by group. Sample sizes reflect the number of participants who provided responses to each question. Percentages represent the proportion of respondents reporting any discrimination experience within each institutional system.

Overall, the reported rates of discrimination were similar between groups (Arabic: 29.2%, Hebrew: 23.8%), although there was variation across institutional systems (Figure 7). In this exploratory sample, respondents to the Arabic survey consistently reported lower discrimination rates in most domains, with the most pronounced differences in housing (Arabic: 10.5% vs. Hebrew: 39.3%) and mental health services (Arabic: 11.8% vs. Hebrew: 32.3%). Education had comparable levels of discrimination (Arabic: 31.6% vs. Hebrew: 33.9%).

When asked to detail whether there was a reference to any characteristic of them on the occasion of discrimination, 14 adolescents in the Hebrew survey provided details. Of them, six mentioned their physical appearance, including: being “overweight,” “religious,” and “white.” Six noted gender (girls and boys). Seven noted nationality; five of them mentioned “being Jewish.” Eight mentioned religion, including being “Jewish,” “religious,” and “secular/non-religious. Five noted ethnic origin (“Ashkenazi,” “religious”). Six mentioned age, one responder detailed: “As if I’m too young for my opinion to be heard.” Among responders to the Arabic survey, 9 participants provided details: One reported physical appearance, specifically “clothing.” Four reported nationality, with one mentioning: “I am an Arab studying in a Jewish school,” and others not detailing further than “nationality.” One responder mentioned ethnic origin, one noted age, and two mentioned “other characteristics without detailing. Preliminary findings suggest that respondents in the Arabic survey reported higher rates of concealing personal characteristics to avoid discrimination (37.5% vs. 24.1%). However, the extremely small sample size does not allow for inferential statistical tests and limits the generalizability of this finding.

#### 3.2.3 Participation and agency

##### 3.2.3.1 Privacy

Overall, 16.7% of respondents to the Arabic survey and 21.8% of the Hebrew survey reported experiencing at least one privacy violation across all measured types. However, here too, we faced substantial sample size limitations and high missingness rates, which compromise analytical validity. Among the limited participants who did respond (Arabic *N* = 11–13 per type, Hebrew: *N* = 57–63), respondents to the Arabic survey reported higher rates of intrusive security checks (36.4% vs. 13.3%) and examination of private materials (27.3% vs. 11.7%) - while showing similar rates for disclosure of personal information (18.2% vs. 17.5%) and invasion of private space (30.8% vs. 30.2%).

##### 3.2.3.2 Freedom of speech

In this exploratory sample, preliminary findings suggest that respondents to the Arabic survey reported feeling less free to express their opinions about the war (*M* = 3.28, *SD* = 2.52, *n* = 18), compared to respondents to the Hebrew survey (*M* = 4.51, *SD* = 2.39, *n* = 92). This difference was also examined using a permutation test (*Z* = 1.96, *p* = 0.050). Again, note that the Arabic survey’s sample size was very small.

When examining consequences adolescents face for sharing their opinions, high missingness rates were observed in both surveys (Arabic: 25–42%, Hebrew: 25–31%). Thus, analyses included the percentage of non-zero responses as a proxy for experiencing consequences. Overall, 25.0% of Arabic survey respondents and 25.7% of Hebrew survey respondents reported experiencing some form of negative consequence. Adolescents in the Hebrew survey reported higher rates of shaming (39.5% non-zero responses) compared to those in the Arabic survey (25.0%), and lower physical assault (4.3% vs. 6.7%). Harm to relationships with peers (20.8, 21.4%) and verbal assault (20.3, 20.0%) showed similar exposure rates between Hebrew and Arabic survey respondent groups, respectively.

About two thirds of the respondents to the Hebrew Survey did not respond to the question “If you do not feel free to share your opinions, what are you afraid of?” of the 37 who did respond, 32% mentioned verbal assaults, 29% fear of shaming, 24% of being boycotted by peers, and 24% mentioned fear of physical assault, 8% of threats, 8% of police violence, and 8% fear of arrest. “Other” reasons included fear of “fights,” “criticism,” “not wanting to talk politics,” and “feelings of shame.” In the Arabic survey, two-thirds of adolescents responded (16 participants). Notably, most of them (81%) mentioned that they were afraid of police violence if they expressed their opinion, and half of them mentioned the fear of arrest. Nineteen percent of those responding to this item in the Arabic survey mentioned fear of physical or verbal assault, and 6% fear of shaming, and of being boycotted by peers. One “other” reason mentioned was the fear of “arresting my parents.”

##### 3.2.3.3 Access to safe information

**Figure 8:**
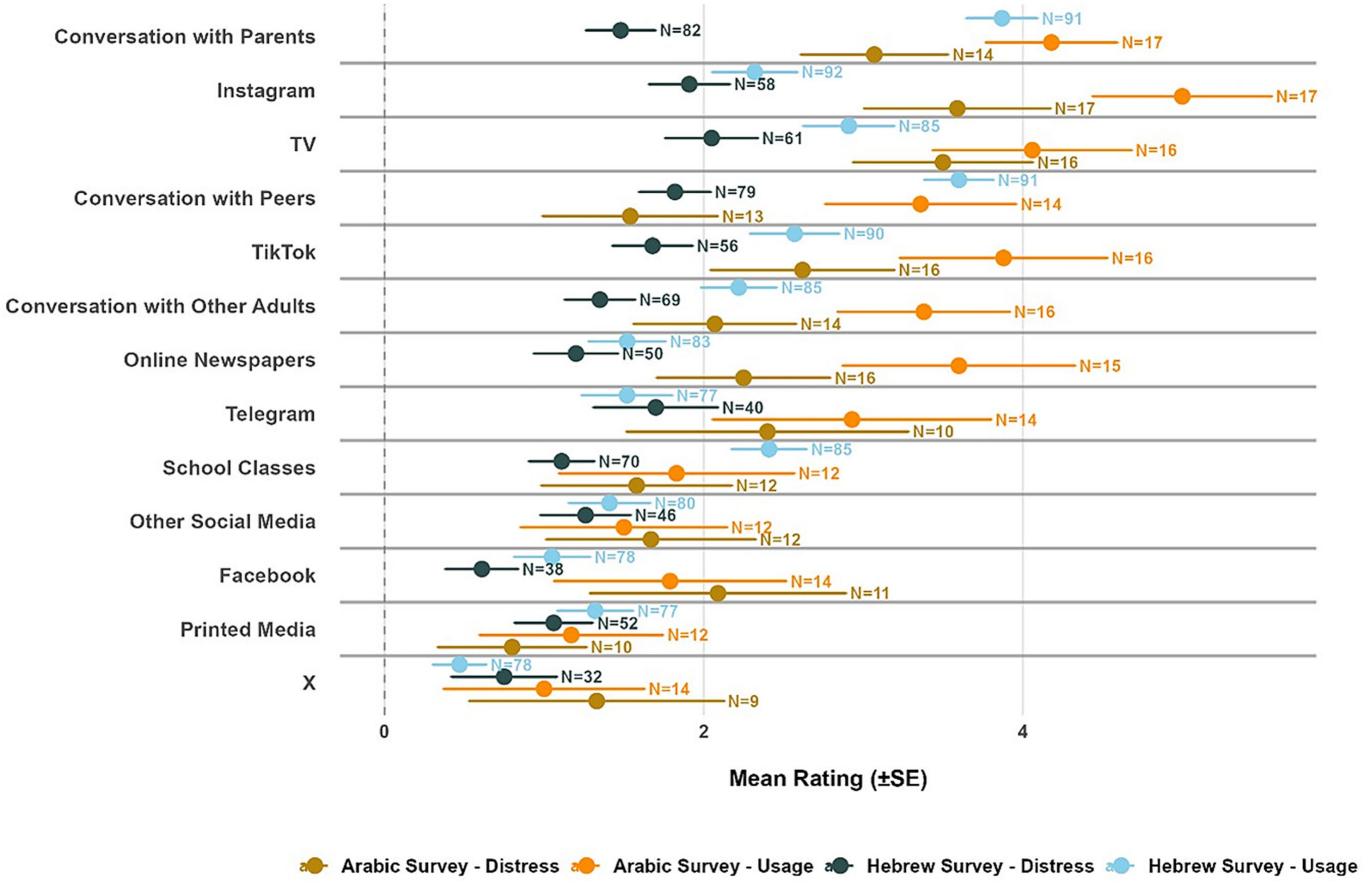
Information source usage frequency and associated distress levels among respondents to the Hebrew and Arabic surveys. *N* reflects the number of participants who provided responses to each question.

Both Arabic and Hebrew survey respondents reported the highest rates of consuming information about the war from conversations with parents (*M*_Arabic_ = 4.18, *M*_Hebrew_ = 3.87), followed by conversations with peers (*M*_Arabic_ = 3.36, *M*_Hebrew_ = 3.60) and television (*M*_Arabic_ = 4.06, *M*_Hebrew_ = 2.91). In this exploratory sample, respondents to the Arabic survey reported higher levels of distress compared to those in the Hebrew survey. Unpaired permutation tests revealed significant group differences for 2 of 13 sources after FDR correction: Instagram (*M*_Arabic_ = 3.59, *SD* = 2.40, *n* = 17 vs. *M*_Hebrew_ = 1.91, *SD* = 1.93, *n* = 58; *p* = 0.040) and conversation with parents (*M*_Arabic_3.07, *SD* = 1.73, *n* = 14 vs. *M*_Hebrew_ = 1.48, *SD* = 1.98; *n* = 82, *p* = 0.040). Other sources of information showed similar directional patterns but did not reach statistical significance after multiple comparison correction (Figure 8).

##### 3.2.3.4 Participation in displacement decisions

Among the four adolescents responding to the Hebrew survey who reported being displaced, reports on participation ranged from 1 (“I received information about the new situation”) to 6 (“The decision was made by me alone”). No responder to the Arabic survey reported being evacuated or displaced.

#### 3.2.4 Well-being

Respondents in the Hebrew and Arabic surveys reported similar levels of well-being (*M*_Hebrew_ = 34.3, *SD* = 4.79; *M*_Arabic_ = 33.0, *SD* = 5.84) with no significant between-groups difference found using a permutation test (*Z* = -1.29, *p* = 0.199). Wave 2 participants (both surveys, *N* = 116) reported higher well-being scores (*M* = 34.3, *SD* = 4.79) compared to Wave I (*N* = 345; *M* = 25.5, *SD* = 4.67), this difference reaching statistical significance (*Z* = 13.53, *p* < 0.001).

## 4 Discussion

The goal of this study was to examine the attainment of adolescents’ rights and needs in Israel during the ongoing multifront war spanning 2023–2025. Through the dual lens of the CRC’s rights-based framework and children’s reports on their own experiences, across the domains of protection, provision, and participation, important patterns emerged that imply both vulnerabilities and resilience, echoing broader international findings that frame children as rights-holders (Machel, 1996; Okorodudu, 1998; Goldhagen et al., 2019).

Assessing children’s *protection* rights, our results indicate that one and a half years after the start of the war, physical protection of children in Israel appears to be generally maintained, and basic needs of adolescents seem to be fulfilled to some extent, including after displacement. Still, about a fifth reported being exposed to some sort of violence, suggesting gaps in the fulfilment of CRC Article 19, with higher rates of exposure to violence reported in the second wave of data collection. A notable theme emerging from the qualitative analysis of text responses was *fear of ‘the other’*. More specifically, many responders to the Hebrew surveys articulated their fear of Arab “places” and people. Although the number of responders to the Arabic survey was very small, the few texts provided aligned with this theme (e.g., “[I avoid] places where I feel unwelcome… that are different from me in language, origin, or religion”). It is important to distinguish between these as individual emotional responses versus broader sociopolitical generalizations. The qualitative data capture individual emotional experiences at a particular point in time and do not necessarily characterize broader social patterns. The finding regarding fear of “the other” must also be contextualized within the specific circumstances of the ongoing war. For Hebrew-speaking respondents, this fear is likely to emerge in the context of the October 7th attacks, ongoing security threats, and media coverage emphasizing threats from Arab militant groups. For Arabic-speaking respondents, fear responses may reflect their experiences as a minority group during heightened intergroup tensions. These patterns, thus, might represent responses to specific traumatic events and conflict circumstances rather than inherent group characteristics. A potential disparity between parents and children in reporting contact with the police warrants further research. Additionally, the anecdotal but notable pattern of more reports of unwanted contact with the police by respondents to the Arabic survey, compared to the Hebrew survey, relative to the total number of respondents, warrants further research about possible violations of children’s right to due process (CRC Articles 37, 40).

One conclusion might be that, although basic and immediate protection rights are generally upheld, children’s sense of safety is impacted in ways that warrant closer inspection. Reported exposure to verbal violence alongside dissatisfaction from schools’ response to violence, reported avoidance and fear, might be significant indicators of unsafety. These were not always evident by reports of safety facilities (e.g., shelters), direct exposure to physical violence or contact with the police, but inner feelings that, even if not presented on the surface or captured in institutional statistics or even parents’ reports, underlie the adolescents’ lives and shape their behaviors. To learn about these senses of unsafety and avoidance, an intentional approach was needed to actively seek the child’s perspective; in other words, to understand the children’s feelings of unsafety, we had to ask them directly. Importantly, the right to protection refers not only to protecting the child’s life and physical and mental health, but also to the right of children and youth to *feel safe* and, thus, also to fulfill other human rights, such as participation (Article 12) and development (Article 6) (Peleg, 2019).

As for children’s *provision* rights, here too, a pattern of reportedly fulfilled immediate basic needs, alongside a reflection on what is not being fulfilled, mainly in the emotional and mental aspects of children’s lives, emerged. In the domain of education, schools are operating and are reportedly relatively adequately protected (from missile attacks). However, the adolescents reported instability and discontinuity in staff. Notably, Arab schools appear to demonstrate more stability, perhaps due to the fact that teaching staff are less affected by the need to serve on reserve duty, and indeed respondents to the Arabic survey reported more school support compared with those in the Hebrew survey. Discrepancies between parents’ and adolescents’ perspectives were implied around the adolescents’ school attendance irregularities: anecdotally, adolescents seem to report more absences than their parents, suggesting a need to further explore a possible gap in the fulfilment of the state’s obligation to “take measures to encourage regular attendance at schools and the reduction of drop-out rates” (CRC Article 28(e)). Such future exploration will need to rely on large enough sample sizes to allow for statistical examination. Of those who chose to share their missing needs, some mentioned the need for both emotional support and more access to information about the situation, and merely to “talk to someone”.

Although there were no extensive reports on missing basic needs or services, our analyses did capture adolescents’ experiences of discrimination, prohibited according to CRC Article 2, for various perceived reasons (“being Jewish,” “being Israeli,” “being Arab,” “being Muslim,” “being secular,” “being religious,” “being Ashkenazi,” “being Mizrachi,” “being Russian,” “being Bedouin,” and more). Perhaps this is a reflection of the fractured Israeli society, as seen in the experiences of youth during a time of instability. Despite this, it appears that most adolescents do not attempt to conceal their characteristics to a significant extent, although it is suggested that those responding to the Arabic survey tended to report more engagement in doing so. The unique challenge of members of Arab minority groups in Israel was expressed in the words of one participant: “I am an Arab studying in a Jewish school.” Importantly, the use of identity labels in reporting discrimination relies on participants’ self-descriptions; these attributions reflect subjective experiences and perceptions, thus, should be situated within a broader interpretive framework that avoids essentializing identity categories. Age was also mentioned as a cause for discrimination by some, with two quotes specifying: “I am not being taken seriously because of my age,” and another: “As if I’m too young for my opinion to be heard.”

What seems to be missing from the present study’s findings is the experiences of discrimination by the adolescents in the Arabic survey, mainly in relation to the well-documented gaps in socio-economic conditions compared with the Jewish majority in education, poverty rates, health indicators, and infrastructure (Daoud et al., 2018). It could be that the adolescents did not report on discrimination due to issues of trust or engagement; it could also be that they are not fully aware of these gaps and what they mean in terms of their rights and needs. This is an additional reason for the further research needed, specifically on this population of Arab adolescents in Israel. Importantly, findings related to reduced freedom of expression among Arabic-speaking adolescents might be interpreted beyond the individual or interpersonal level to reflect broader structural, legal, or sociopolitical constraints on the rights of children from minority groups in Israel. This important question should be directly addressed in future research.

The study also highlights some threats to children’s agency and participation rights. A notable number of adolescents experienced some degree of privacy violation, protected under CRC Article 16, which they may have encountered while regularly using joint shelters and during security checks, experiences that might compromise their dignity and autonomy. As to children’s right to freedom of expression (CRC Article 13), parents tend to think that their children are freer to express their opinions about the war than adolescents actually report. Additionally, parents often underestimate the consequences that adolescents experience for publicly sharing their opinions, which in the Hebrew surveys mainly included fear of and consequences of shaming, as well as harm to relationships with peers. Notably, respondents to the Arabic survey reported feeling less comfortable expressing their opinion about the war, and when asked what they feared, a common response was fear of police violence and even arrest, reflecting a climate of reduced freedom of speech. These findings join earlier claims that conflict disproportionately threatens the voices of children in minority groups (Okorodudu, 1998; Goldhagen et al., 2019). In fact, the respondents to the Arabic survey in this study were not just a minority group, but also likely to experience a spectrum of complexities as Arabs in the midst of a war in Israel, and in the broader context of the Israeli-Palestinian conflict. It can be speculated that the atmosphere of reduced freedom of speech, alongside potential issues of identity and belonging (also protected under CRC Articles 8 and 29(c)), also contributed to their higher reported distress from consuming information on the war from social media (specifically Instagram) and from conversations with parents. However, despite these constraints, the majority of participants reported being able to express their opinions freely and having access to information with relatively low distress levels.

Although the number of adolescents who were displaced from their home was small in our sample and did not allow for statistical analyses, a qualitative examination suggests that those who chose to report their involvement in decision-making regarding displacement experienced high levels of involvement and participation, with reports on getting information, making decisions and receiving adults support in deciding on school and afterschool activities, and even the family accommodations. Parents’ reports seem to corroborate this pattern of participation. Of course, this anecdotal report regarding the fulfillment of CRC Article 12 on participation in family decisions among displaced children does not translate to a generalized finding, and we suggest that further research specifically examine the protection, provision, and participation rights of displaced children.

The findings echo an ecological perspective on children’s rights (Gal, 2017, 2020), which highlights the importance of the “circles” around the child in fulfilling their rights, especially participation rights. First, the parents are most immediate and significant. The present results indicate that parents are a central source of information for their children and that they can serve as agents of participation, for example, in the extreme case of displacement. Another important circle in children’s lives is their social environment, and some adolescents indeed mentioned that they fear peer boycott or harm to relationships if they express their views, demonstrating how their right to participation is affected by their eco-system. Finally, the safety issues that some children described may indicate how the external circle of the broader society affects the children’s ability to fulfil their rights to participation enshrined in Articles 12, 13, 14, and 15.

Despite some discrepancies in perspectives (for example, parents’ tendency to overestimate privacy violations and freedom of expression of their children), parents generally read their children’s emotional state and needs well, as was most notably demonstrated in their reporting of their children’s wellbeing. Parents also assessed exposure to information as more distress-evoking than what adolescents reported experiencing. At the same time, adolescents reported that the primary source of information about the war was their parents, and a relatively safe source. It is possible that while some parents attempt to shield their children from perceived distress by reducing exposure to some information sources, adolescents not only rely more on conversation with parents, but also may feel the need for more access to information, a protected right under CRC Article 17, as reflected in some of the text responses. The observed discrepancies between adolescents’ and parents’ reports warrant further consideration. Several potential mechanisms may explain these differences. First, adolescents may withhold information from parents about sensitive experiences (e.g., violence exposure, discrimination, consequences of expressing opinions) due to concerns about parental worry or potential restrictions on their autonomy. Second, parents may underestimate their children’s experiences due to limited awareness of adolescents’ social interactions outside the home, particularly in school and peer contexts. Third, parents and adolescents may use different reference points when evaluating experiences. For example, parents may compare to their own childhood experiences or broader population norms, while adolescents compare to their peers’ experiences. Fourth, adolescents may be more attuned to subtle forms of discrimination that parents do not observe. These possible explanations suggest that both perspectives provide valuable but complementary information, with adolescents’ reports offering insights into experiences that may not be visible to parents, particularly in domains involving peer relationships, school environments, and internal experiences such as fear of expressing opinions.

The exploratory preliminary comparison between the Hebrew and the Arabic surveys indicated some between-group differences regarding parents’ role in the attainment of their children’s rights. First, in the Arabic survey, significantly more verbal assault in the home environment was reported by the adolescents compared to the Hebrew survey. Second, while parents were reported to be the primary source of information about the war in both groups, adolescents in the Arabic survey reported significantly more distress from such conversations with their parents compared to the Hebrew survey. While caution is needed in interpreting these preliminary findings due to some limitations of the present Arabic sample (mainly its small size and convenience recruitment method), they align with previous literature addressing the unique challenges of disadvantaged minority groups in the context of parenting. Previous studies indicated that socio-political stress and chronic ethnonational minority stress are associated with parents’ psychological distress, which in turn is linked to elevated risk of family violence such as child abuse and neglect, among Palestinian parents in Israel (Zedan, 2024; Zedan and Haj-Yahia, 2023, 2024). Similarly, Palestinian adolescents in East Jerusalem described collective and intergenerational transmission of stress, sustaining a persistent emotional climate of fear and insecurity; adolescents reported that parental fears and family talk about ongoing political threats circulate at home, transmitting stress across generations, and in some families, fear of repercussions leads to silencing or avoidance of such discussions (Zedan, 2025). While patterns in our findings align with previous literature on minority stress and socio-political challenges, we emphasize again that these are preliminary and require cautious interpretation. The very small Arabic sample size (*n* = 24) and convenience sampling method limit our ability to draw firm conclusions about group differences. The observed patterns may reflect multiple factors beyond socio-political context, including sample characteristics, response patterns, or other unmeasured variables. Future research with larger, more representative samples is needed to substantiate these preliminary, exploratory findings and better understand the mechanisms underlying any group differences.

In the second “circle,” the child’s social environment, the present study’s findings highlight significant challenges in the education system in maintaining children’s rights and in addressing their needs during war. When data were collected for this study, the war had been ongoing for more than a year. According to participants’ reports, most schools were operating and had adequate safety facilities, seemingly attaining the fundamental rights to protection and provision. However, the finding of notable staff instability, alongside repeated text responses articulating the adolescents’ need for more help, support, and understanding in schools, suggests that these rights are not being fully fulfilled. Adolescents expressed both their need for more support in areas of study and learning, and a call for emotional support and help to address their mental health, with some comments being particular and expressing an immediate need (e.g., “[I need] a psychologist in school”). As most participants reported schools to be adequate to protect children in the event of a missile attack (i.e., have shelters or safety facilities), a notable percentage of adolescents and parents did express concerns about shelters in schools, to some degree. Schools were also reported to be a place where adolescents are exposed to verbal and sometimes physical violence, and both adolescents and parents reported low satisfaction with the way schools are dealing with violence to protect children. These findings resonate with UNESCO (2011), which documented school closures, teacher displacement, and destroyed infrastructure as recurring barriers to learning and wellbeing in conflict settings worldwide. They also echo the Machel Report’s identification of education as both a provision and protection right critical to children’s resilience (Machel, 1996).

Adolescents’ rights to participation in schools, and specifically in times of war, are another important area. We did not specifically target the school setting separately from other environments in the adolescent’s life when inquiring about participation and freedom of expression. However, the school is a central arena in their life for social interactions with peers and meaningful adults. Thus, when our findings suggest that adolescents are sometimes afraid to express their opinions and experience consequences, such as shaming and harm to their relationships with peers, this is also likely related to schools. Notably, we also found that parents tend to underestimate their children’s fear of and experiences of such consequences. Some adolescents clearly articulated in their text responses their need for schools to be a source for information and discussion, and “to be talked to more openly in school”, specifically “about the situation”. It also appears that as time passes and the war continues, the fulfillment of educational needs and rights under CRC Articles 28 and 29 does not improve, but rather worsens, as indicated by the lower fulfillment of educational needs reported in our second wave of data collection compared to the first wave. The alarming finding of a reduction in the fulfillment of adolescents’ educational needs and rights calls not only for further research but also for immediate action.

The schooling system in Israel operates mainly separately for the majority, mostly Jewish, Hebrew-speaking children, and for the children from Arab minorities. This separation relates to the attainment of children’s rights and needs in the “outer” circle, which is the broader society. Our findings, which include avoidance and fear of the ‘other’ (mainly in the Hebrew surveys) and a sense of limited freedom of expression (mainly in the Arabic survey), suggest limited participation in society among diverse groups. It can be speculated that such a disconnection between groups becomes more pronounced during times of war. Adolescents in our Hebrew samples expressed explicit fear and avoidance of “Arab places” and “Arab people.” Considering the unprecedented events of the October 7th carnage, and specifically the abduction of youth, children, and toddlers, alongside a routine of daily missile attacks from Arab militant groups, fear seems to be a natural emotional response. At the same time, our respondents to the Arabic survey, experiencing the same threats of terror and the same missile attacks, experienced heightened distress when consuming information about the war; they are probably more exposed to the impact of the war on children and youth in Gaza through media and social media in Arabic, parents, and perhaps even relatives. These adolescents feel less free to express their opinions and articulate explicit fear of police violence and arrest if they do so. Imagine a child from each group; the two of them probably do not attend the same school, and are unlikely to interact in community activities or public spaces. One can assume that with time discrepancies, fear, and avoidance will only grow, limiting children’s protection rights under the CRC (such as the right to safety and feeling safe), provision rights (non-discrimination), and meaningful participation rights as enshrined in Articles 12 and 13, unless action is taken to bring these two groups together. The ongoing war presents a literal obstacle to such possible attempts, but it also highlights the importance of this aim as a sore point.

Lastly, we note the somewhat optimistic, though perhaps paradoxical, finding of improved well-being levels among adolescents in the second wave of data collection compared to the first wave. This may reflect processes of adaptation or normalization, as the war continued. It could also point to a possible process of recovery from an initial tremendous shock and trauma. Similar processes have been noted in international reviews of children in war zones, where resilience and adjustment often coexist with vulnerability (Slone and Mann, 2016). However, alternative explanations must also be considered: the improved well-being could reflect sample composition differences between waves, as participants of the two waves may differ in unmeasured characteristics (e.g., resilience, social support, or baseline wellbeing). Selection bias is also possible, as participants who chose to participate in Wave II may have been those experiencing better adjustment. Additionally, the timing difference may have captured different phases of the conflict or different seasonal factors. Future longitudinal research tracking the same individuals over time would help distinguish between true improvement and sample composition effects. Future studies should also directly investigate whether children’s well-being is associated with other forms of children’s rights attainment and violations.

Despite its importance and novelty, this study has some notable limitations that should be considered when interpreting the findings and drawing conclusions. First, the cross-sectional design limits the ability to draw causal inferences. Additionally, the two waves of data collection employed different samples; therefore, any speculations about changes over time should be made with due caution. The second main limitation is the small sample obtained for the Arabic survey, alongside the convenience sampling approach, which may introduce selection bias. Specifically, this sample employed only Muslim participants and thus does not capture the full spectrum of Arabic-speaking minorities in Israel, such as Christians and Druze. The consequences of non-representative sampling warrant emphasis: selection bias may mean our findings underestimate or overestimate prevalence, and caution is needed when generalizing to broader populations. We note that ongoing work is being conducted using the Arabic survey, and the results reported here are therefore preliminary and exploratory in nature. Third, although we aimed for a representative sample to reflect the highly varied and heterogeneous Israeli society, we cannot be certain that this goal was achieved in the Hebrew survey as well. In future studies, it is recommended that more detailed demographic information be obtained, including participants’ socio-economic status and whether they belong to minority groups (e.g., immigrants, refugees, or individuals with disabilities). We also had relatively low representation of adolescents who were displaced during the war, suggesting we probably did not have enough representation of children experiencing the October 7th terror attack firsthand. The degree to which these can be generalized is extremely limited given the small sample. We also cannot assure that our sample included adolescents who lost a parent or a sibling, or were injured.

We were limited in our ability to collect such data, as well as data of an extremely sensitive nature, such as physical violence in the home or sexual assaults, due to ethical guidelines and precautionary measures. Undoubtedly, such data is important, specifically in times of instability. However, we could not compromise the safety of participants, and the present design did not allow the collection of such data. We also recognize that high missingness rates in our current data, particularly in the Wave II Arabic sample, may reflect sensitivity of topics or cultural factors. While we report missingness rates by domain and group, the small sample sizes precluded formal statistical tests of missingness patterns. Future research should investigate factors contributing to missingness in such sensitive research, particularly among minority populations during conflict. High missingness rates for sensitive topics may reflect social desirability bias or response avoidance, meaning our findings may underestimate the true prevalence of negative experiences. More broadly, future research should increase efforts to recruit participants from marginalized populations and to maximize cultural and linguistic adaptations during participant recruitment.

This pioneering study represents the first systematic assessment of children’s rights attainment during war using a CRC-based framework. Given its exploratory nature, we acknowledge that a formal psychometric validation is needed for the novel questionnaire items in future studies, relying on larger sample sizes. The questionnaire was designed as a rights-based assessment tool that maps directly to CRC articles, and the urgent context of documenting children’s experiences during an ongoing conflict guided our approach. In addition to ensuring face validity through a process of iterative development of the questionnaire, we employed triangulation through multiple information sources. These included combining adolescents’ and parents’ reports, and incorporating quantitative and qualitative data, to strengthen the validity of our findings regarding children’s rights attainment. Future research with larger samples should examine the structural coherence and reliability of the questionnaire items to further validate this novel assessment approach.

Finally, we recognize the impact of the ongoing war on children outside of Israel, and specifically in Gaza. While examining the impact of war on children and youth in Gaza is beyond the scope of this study, and was not applicable for an Israeli research team as the war is ongoing, we clearly state, as others, that such research is of great importance and urgency (e.g., Alibwaini and Thabet, 2019; Bessell and O’Sullivan, 2026). We hypothesize, though, that the findings of the present study focusing on adolescents in Israel provide important insight into the impact of war on children’s rights and needs in all comparable contexts.

Notwithstanding these limitations, this study makes a significant contribution to the broader understanding of how war impacts adolescents in a novel methodological and epistemological manner. By employing an interdisciplinary framework that combines a normative rights-based analysis with an empirical examination of children’s reports on the attainment of their rights and needs, this work presented findings emphasizing often-overlooked impact of war on children such as freedom of speech, equality, access to safe information, and participation, and highlight the impacts of prolonged war on the sense of safety and agency beyond immediate needs. To address gaps in protection, provision, and participation rights identified in this study, a few actionable recommendations and policy implications regarding children and adolescents in times of war might be: strengthening school-based violence prevention programs, ensuring educational continuity and mental health support services, implementing regular child-centered monitoring of rights attainment and ensuring representation of minority groups in policy development, and creating safe spaces for children and youth to express their views.

## Data Availability

The raw data supporting the conclusions of this article will be made available from the authors after anonymization, on reasonable request.

## Footnotes

2 Hamas is a designated terrorist organization by the United States of America: https://www.state.gov/foreign-terrorist-organizations; The European Union: https://www.consilium.europa.eu/en/policies/sanctions-against-terrorism/#list, the United Kingdom: https://www.gov.uk/government/publications/proscribed-terror-groups-or-organisations--2/proscribed-terrorist-groups-or-organisations-accessible-version, Canada: https://www.canada.ca/en/public-safety-canada/news/2025/12/government-of-canada-lists-four-new-terrorist-entities0.html, Australia: https://www.nationalsecurity.gov.au/what-australia-is-doing/terrorist-organisations/listed-terrorist-organisations, Israel: https://nbctf.mod.gov.il/en/Minister%20Sanctions/Designation/Pages/downloads.aspx, and more.

3 The term “massacre” was used to describe the October 7th attack by multiple UN and EU officials: Borrell, J. (2023, November 22). Israel/Gaza: Speech by High Representative/Vice-President Josep Borrell at the EP Plenary. European External Action Service. https://www.eeas.europa.eu/eeas/israelgaza-speech-high-representativevice-president-josep-borrell-ep-plenary_en; United Nations (2023, October 18). World “at brink of abyss that could change trajectory of Israeli-Palestinian conflict”, UN Middle East Coordinator warns Security Council (Press release SC/15451). UN Meetings Coverage and Press Releases. https://press.un.org/en/2023/sc15451.doc.htm; von der Leyen, U. (2023, November 9). Speech by President von der Leyen at the International Humanitarian Conference for the Civilian Population in Gaza. European Commission. https://enlargement.ec.europa.eu/news/speech-president-von-der-leyen-international-humanitarian-conference-civilian-population-gaza-2023-11-09_en.
