# Supplementary material for: Children’s rights and needs during war: the case of adolescents in Israel
Source: Front Psychol. 2026 Mar 2;17:1719621. doi: 10.3389/fpsyg.2026.1719621 (PMC12989495; doi:10.3389/fpsyg.2026.1719621)
Supplement: Supplementary file 1 [file Data_Sheet_1.pdf]

Supplementary Table 1: Children's Rights During War: A CRC Analysis

Domain	Article No. and Title	Meaning in the Current Context	Examples of Specific questions
**Protection**	Article 3 – Best Interests of the Child	Sufficient staffing and funding must be provided to ensure children's well-being in all domains.	"To what extent do you feel that your various needs as a child are being met?"<br>● Education<br>● Physical safety<br>● Safety in cyberspace<br>● Mental health support<br>● Health<br>● Health and safety for my family members<br>For displaced children: "To what extent are the activities you are used to (for example, clubs) available in your current place of residence?"
	Article 19, Protection from Abuse and Neglect	Protection of children from harm by caregivers, educators, and others; children's sense of being safe from harm.	"In the past two months, have you been directly harmed by physical or verbal violence in any of the following ways?"<br>(1) In an educational institution<br>(2) In a public space<br>(3) In another place<br><br>"Are there places you are afraid to go to?"
	Article 22, Rights of Refugee Children	Providing optimal responses for displaced or fleeing children in accordance with their best interests.	For displaced children: "To what extent are there adequate physical conditions in your current place of residence (for example: a protected space, a sufficient number of rooms for everyone, a kitchen, a living room, beds for everyone)?"
	Articles 37, 40, Protection from Torture and Cruel Punishment; due process	Preventing arbitrary and prolonged detention of youth, and ensuring fairness in police investigation and treatment.	"Have you had any unwanted contact with the police recently?"<br>"If you answered yes to the previous question, was the treatment you received fair?"
	Article 2 – Equality and	Allocation of appropriate and equal resources to support	"Below is a list of 'services' provided by the state (education, health, welfare,

**Provision**	Non-Discrimination	children and families affected by the war in health, mental health, education, housing, rehabilitation, and leisure.	security). You may not have had any contact with representatives of these services, but you might have. If you did, has it happened in the past two months that representatives of these services discriminated against you?"
	Articles 6, 26, 27 – Right to Life, Survival, Development, and Adequate Living Conditions and social security	Adequate developmental services, including education, mental health, leisure, and family support	"Which services and/or activities do you think you should have received but did not?"<br>"To what extent do you feel that your family lacks basic necessities? (For example, food, clothing, housing, medical services, etc.)"
	Article 24, 39, Right to Health and a Healthy Environment, and to Rehabilitation	Ensuring proper health and mental health services for all children.	"To what extent do you feel that your various needs as a child are being met?"<br>(1) Mental health support<br>(2) Health<br>(3) Health and safety for my family members
	Articles 28, 29, 31 Right to Accessible and Quality Education including leisure and social activities	Guaranteeing inclusive, quality, safe education during the war for all children in Israel; allocating resources for emotional and social needs including time and space for leisure and play; ensuring continuity in education while addressing war-related needs.	"Is your school currently operating?"<br>Do you feel that any of the educational needs listed below have been lacking for you in the past two months?"<br>(1) Leisure activities<br>(2) Tutoring lessons<br>(3) Youth movement activities<br>(4) Preparation for matriculation exams<br>(5) Enrichment for gifted and talented students<br>(6) Homeroom teacher lessons<br>(7) Social and team-building activities
	Articles 12 – Right to Participation in Decisions	Involving children in decision-making processes on issues affecting their lives.	For displaced children: "For each decision, respond in the way that seems most accurate to you according to this scale:<br>0 – I was not involved at all<br>1 – I received information and an explanation about the new

**Participation**			situation<br>2 – I expressed my opinion, but it was not taken into account<br>3 – I was consulted, but the adults made the decision<br>4 – There was a joint discussion, and the decision was made together by me and the adults<br>5 – There was a joint discussion, and I made the decision<br>6 – The decision was made by me alone<br>7 – I initiated the decision, and the adults joined and supported". The issues relate to:<br>(1) What will be our new place of residence<br>(2) What will be my new educational setting<br>(3) What will be my new leisure activities<br>(4) Other (please specify)
	Articles 13, 14, 15 - Freedom of Speech, Thought, Conscience, Religion, and Association	Guaranteeing freedom of expression and preventing hate speech and violent reactions to children’s views.	"Do you feel free to share your opinions and feelings about the war/situation in public spaces?"<br>"Have you experienced a physical [or verbal] assault by another person due to expressing your opinion in public spaces in the past two months?"
	Article 16, Right to Privacy	Avoiding the publication of harmful photos or videos, protecting their physical privacy	"Below are different areas related to your privacy. To what extent have there been violations in each of them over the past two months?"<br>(1) Disclosure of personal details (e.g., diary)<br>(2) Entry into your private space (e.g., your room) in a way that violates your privacy<br>(3) Inspection of your personal belongings inside or at the entrance to the school<br>(4) Intrusive security checks

	Article 17, Access to Safe Media	Ensuring delivery of child-appropriate information. Providing education and prevention regarding exposure to harmful content.	"To what extent has exposure to content and information caused you to feel distress or difficulty recently?" (Following a list of various types of information sources, including social media)
	Article 30, Rights to Culture, Language, and Religion of Minorities	Ensuring the safety of all children while maintaining their identity,, given the rising hostility based on religion, nationality, or ethnicity.	"Do you try to hide characteristics that could reveal your nationality, religion, origin, or any other characteristic?"
