# Supplementary material for: Children’s rights and needs during war: the case of adolescents in Israel
Source: Front Psychol. 2026 Mar 2;17:1719621. doi: 10.3389/fpsyg.2026.1719621 (PMC12989495; doi:10.3389/fpsyg.2026.1719621)
Supplement: Supplementary file 2 [file Data_Sheet_2.pdf]

Table 1 S

***Protection** Data Quality: Parent and Child Comparisons*

Variable	N Valid (P/C)	% Missing (P/C)	% Zeros (P/C)	Gender Test *p* (P/C)	Geography Test *p* (P/C)
Physical violence - School	245/241	19.9%/21.2%	90.2%/92.9%	.389/.991	.149/.449
Physical violence - Public	240/239	21.6%/21.9%	92.9%/97.1%	.430/.267	.055/.288
Physical violence - Other	161/174	47.4%/43.1%	95.7%/96.6%	.373/.655	.038/.067
Harassment/Threats - Home	230/233	24.8%/23.9%	94.8%/95.7%	.402/.433	.070/.854
Harassment/Threats - School	234/239	23.5%/21.9%	82.9%/84.1%	.659/.780	.096/.859
Harassment/Threats - Community	218/224	28.8%/26.8%	97.2%/98.7%	.242/.554	.035/.195
Harassment/Threats - Public	222/230	27.5%/24.8%	90.1%/92.2%	.427/.371	.013/.572
Harassment/Threats - Other	161/167	47.4%/45.4%	96.9%/98.8%	.421/.559	.013/.569
Help-seeking satisfaction (conditional)	37/32	36.2%/34.7%	40.5%/37.5%	.224/1.000	.063/.693
Educational needs	—/290	—/5.2%	—/3.4%	—/.516	—/.967
Physical safety	—/275	—/10.1%	—/2.5%	—/.351	—/.764
Social network safety	—/240	—/21.6%	—/3.8%	—/.895	—/.289
Mental health support	—/152	—/50.3%	—/13.8%	—/.274	—/.569
Health needs	—/244	—/20.3%	—/3.3%	—/.123	—/.339
Family health & safety	—/255	—/16.7%	—/2%	—/1.000	—/.997
Information access	—/252	—/17.6%	—/0.8%	—/.929	—/.669
Protection from harm by caretakers	—/177	—/42.2%	—/4%	—/.887	—/.344
Accommodation/permanent residence	—/221	—/27.8%	—/1.8%	—/1.000	—/.797

Note.

P = Parent; C = Child. Values before the slash represent parent data, values after the slash represent child data. Em dashes (—) indicate data not collected for that group. *p*-values shown for gender and geography chi-square tests.
