# Supplementary material for: Children’s rights and needs during war: the case of adolescents in Israel
Source: Front Psychol. 2026 Mar 2;17:1719621. doi: 10.3389/fpsyg.2026.1719621 (PMC12989495; doi:10.3389/fpsyg.2026.1719621)
Supplement: Supplementary file 3 [file Data_Sheet_3.pdf]

Table S1-a

***Protection Data Quality**: Hebrew and Arabic Survey Sample Comparisons*

Variable	N Valid (A/H)	Missing N (A/H)	Missing % (A/H)	Zero % (A/H)
Physical Violence - School	13/75	11/26	45.8%/25.7%	50%/68.3%
Physical Violence - Public	14/74	10/27	41.7%/26.7%	50%/67.3%
Physical Violence - Other Places	7/43	17/58	70.8%/57.4%	25%/40.6%
Verbal Harassment - At Home	13/75	11/26	45.8%/25.7%	37.5%/65.3%
Verbal Harassment - School	14/75	10/26	41.7%/25.7%	41.7%/63.4%
Verbal Harassment - Community	13/69	11/32	45.8%/31.7%	50%/65.3%
Verbal Harassment - Public	12/74	12/27	50%/26.7%	37.5%/60.4%
Verbal Harassment - Other Places	4/40	20/61	83.3%/60.4%	16.7%/37.6%
Institutional Response (among those with any violence)	4/17	2/9	33.3%/34.6%	16.7%/23.1%
Avoiding Places Due to Fear	15/84	9/17	37.5%/16.8%	25%/49.5%
Education	20/95	4/6	16.7%/5.9%	4.2%/3%
Physical Safety	19/82	5/19	20.8%/18.8%	4.2%/5%
Social Media Safety	19/71	5/30	20.8%/29.7%	4.2%/4%
Mental Health	17/50	7/51	29.2%/50.5%	4.2%/11.9%
Health	17/74	7/27	29.2%/26.7%	4.2%/2%
Family Health	16/76	8/25	33.3%/24.8%	0%/1%
Information Access	15/78	9/23	37.5%/22.8%	0%/2%
Protection from harm by caretakers	13/58	11/43	45.8%/42.6%	4.2%/5%
Permanent Accommodation	16/63	8/38	33.3%/37.6%	0%/3%

Note.
A = Arabic survey sample; H = Hebrew survey sample. Values before the slash represent Arabic survey sample data, values after the slash represent Hebrew survey sample data. N Valid calculated as total N minus missing N. Zero % calculated from valid N only.
