# Supplementary material for: Children’s rights and needs during war: the case of adolescents in Israel
Source: Front Psychol. 2026 Mar 2;17:1719621. doi: 10.3389/fpsyg.2026.1719621 (PMC12989495; doi:10.3389/fpsyg.2026.1719621)
Supplement: Supplementary file 4 [file Data_Sheet_4.pdf]

Table 2 S

Provision *Data Quality: Parent and Child Comparisons*

Variable	N Valid (P/C)	% Missing (P/C)	% Zeros (P/C)	Gender Test *p* (P/C)	Geography Test *p* (P/C)
General needs deprivation	—/261	—/14.7%	—/70.9%	—/1.000	—/.167
School operating	—/304	—/0.7%	Binary	NA/1.000	NA/.284
Protection concerns	305/305	0.3%/0.3%	Binary	1.000/.976	.246/.169
Staff changes	—/305	—/0.3%	Binary	NA/.976	NA/.169
Attendance irregularities	278/276	9.2%/9.8%	Binary	.554/.429	.508/.746
Discrimination - Education	260/232	15%/24.2%	70.8%/61.6%	.427/.433	.364/.108
Discrimination - Health	251/193	18%/36.9%	76.1%/73.1%	.600/.971	.260/.021
Discrimination - Welfare	254/99	17%/67.6%	76%/70.7%	1.000/.721	.073/.016
Discrimination - Housing	243/113	20.6%/63.1%	81.9%/69%	.440/.527	.094/.021
Discrimination - Police	238/93	22.2%/69.6%	80.7%/71%	.143/.555	.109/.007
Discrimination - Mental Health	240/94	21.6%/69.3%	73.3%/72.3%	.205/.225	.025/.027
Hide characteristics	—/260	—/15%	—/77.7%	—/.830	—/.561

Note.

P = Parent; C = Child. Values before the slash represent parent data, values after the slash represent child data. Em dashes (—) indicate data not collected for that group. NA indicates test not applicable. *p*-values shown for gender and geography chi-square tests.
