# Supplementary material for: Children’s rights and needs during war: the case of adolescents in Israel
Source: Front Psychol. 2026 Mar 2;17:1719621. doi: 10.3389/fpsyg.2026.1719621 (PMC12989495; doi:10.3389/fpsyg.2026.1719621)
Supplement: Supplementary file 5 [file Data_Sheet_5.pdf]

Table S2-a

***Provision Data Quality**: Hebrew and Arabic Survey Comparisons*

Variable	N Valid (A/H)	Missing N (A/H)	Missing % (A/H)	Zero % (A/H)
Basic Needs Deprivation	19/76	5/25	20.8%/24.8%	68.4%/71.1%
School Support	20/99	4/2	16.7%/2%	5%/5.1%
Discrimination - Education	19/62	5/39	20.8%/38.6%	68.4%/66.1%
Discrimination - Health	20/61	4/40	16.7%/39.6%	85%/77%
Discrimination - Welfare	20/31	4/70	16.7%/69.3%	75%/71%
Discrimination - Housing	19/28	5/73	20.8%/72.3%	89.5%/60.7%
Discrimination - Police	17/35	7/66	29.2%/65.3%	82.4%/71.4%
Discrimination - Mental Health	17/31	7/70	29.2%/69.3%	88.2%/67.7%
Concealing Characteristics	16/79	8/22	33.3%/21.8%	62.5%/75.9%

Note.
A = Arabic Survey; H = Hebrew Survey. Values before the slash represent Arabic survey sample data, values after the slash represent Hebrew survey sample data. N Valid calculated as total N minus missing N. Zero % calculated from valid N only.
