# Supplementary material for: Children’s rights and needs during war: the case of adolescents in Israel
Source: Front Psychol. 2026 Mar 2;17:1719621. doi: 10.3389/fpsyg.2026.1719621 (PMC12989495; doi:10.3389/fpsyg.2026.1719621)
Supplement: Supplementary file 6 [file Data_Sheet_6.pdf]

Table S3

Data Quality for ***Participation*** *(Privacy & Freedom of Speech), and* ***Well-Being*** *Variables, Parents' and Children's Responses Comparison*

Section	Variable	N Valid (P/C)	% Missing (P/C)	% Zeros (P/C)	Gender Test *p* (P/C)	Geography Test *p* (P/C)
Privacy	Disclosure of Personal Information	210/179	31.4%/41.5%	71.4%/87.7%	.921/.881	.359/.095
Privacy	Invasion of Private Space	204/201	33.3%/34.3%	76%/75.6%	.882/.474	.138/.033
Privacy	Examination of Private Materials	192/185	37.3%/39.5%	78.1%/85.9%	.999/.522	.027/.148
Privacy	Intrusive Security Checks	173/171	43.5%/44.1%	81.5%/91.8%	.816/.725	.028/.026
Freedom of Speech	Opinion Sharing Freedom	298/285	2.6%/6.9%	3.7%/13%	.955/1.000	.619/.493
Freedom of Speech	Consequence: Physical Assault	237/236	22.5%/22.9%	96.6%/94.9%	.662/.587	.660/.189
Freedom of Speech	Consequence: Verbal Assault	272/233	11.1%/23.9%	88.6%/82.8%	.362/.906	.191/.150
Freedom of Speech	Consequence: Peer Relationship Harm	230/234	24.8%/23.5%	90.4%/81.6%	.991/.990	.077/.006
Freedom of Speech	Consequence: Shaming	231/238	24.5%/22.2%	90.5%/76.9%	.643/.578	.089/.063
Well-Being Items	Emotion item 1	306/305	0%/0.3%	2%/2.3%	—	—
Well-Being Items	Emotion item 2	303/305	1%/0.3%	0.7%/1.6%	—	—
Well-Being Items	Emotion item 3	305/305	0.3%/0.3%	26.9%/32.1%	—	—
Well-Being Items	Emotion item 4	306/304	0%/0.7%	42.8%/49%	—	—
Well-Being Items	Emotion item 5	305/305	0.3%/0.3%	0.7%/1.6%	—	—
Well-Being Items	Emotion item 6	306/305	0%/0.3%	1%/0.3%	—	—
Well-Being Items	Emotion item 7	306/304	0%/0.7%	1.3%/1%	—	—
Well-Being Items	Emotion item 8	306/305	0%/0.3%	1.3%/1.6%	—	—
Well-Being Items	Emotion item 9	305/305	0.3%/0.3%	0.3%/2.3%	—	—
Well-Being Items	Emotion item 10	304/305	0.7%/0.3%	2%/3%	—	—

Note.
P = Parent; C = Child. Values before the slash represent parent data, values after the slash represent child data. Em dashes (—) indicate data not applicable. Due to small amount of missing values (mostly between 0-3%), statistical testing not reported for well-being items. *p*-values shown for gender and geography chi-square tests.
