# Supplementary material for: Children’s rights and needs during war: the case of adolescents in Israel
Source: Front Psychol. 2026 Mar 2;17:1719621. doi: 10.3389/fpsyg.2026.1719621 (PMC12989495; doi:10.3389/fpsyg.2026.1719621)
Supplement: Supplementary file 7 [file Data_Sheet_7.pdf]

Table S3-a

Privacy, Freedom of speech, and Well-Being Data Quality: Arabic and Hebrew Survey Sample Comparisons

Variable	N Valid (A/H)	Missing N (A/H)	Missing % (A/H)	Zero % (A/H)
Privacy: Type 1	11/57	13/44	54.2%/43.6%	81.8%/82.5%
Privacy: Type 2	13/63	11/38	45.8%/37.6%	69.2%/69.8%
Privacy: Type 3	11/60	13/41	54.2%/40.6%	72.7%/88.3%
Privacy: Type 5	11/60	13/41	54.2%/40.6%	63.6%/86.7%
Freedom: Feeling Free	18/92	6/9	25%/8.9%	22.2%/7.6%
Freedom: Physical Assault	15/70	9/31	37.5%/30.7%	93.3%/95.7%
Freedom: Verbal Assault	15/74	9/27	37.5%/26.7%	80%/79.7%
Freedom: Boycott	14/72	10/29	41.7%/28.7%	78.6%/79.2%
Freedom: Shaming	16/76	8/25	33.3%/24.8%	75%/60.5%
Well-being: Emotion Score	19/97	5/4	20.8%/4%	0%/0%

Note.
A = Arabic survey sample; H = Hebrew survey sample. Values before the slash represent Arabic survey sample data, values after the slash represent Hebrew survey sample data. Missing N calculated as Total N minus N Valid. Zero % calculated from valid N only.
