# Supplementary material for: Children’s rights and needs during war: the case of adolescents in Israel
Source: Front Psychol. 2026 Mar 2;17:1719621. doi: 10.3389/fpsyg.2026.1719621 (PMC12989495; doi:10.3389/fpsyg.2026.1719621)
Supplement: Supplementary file 8 [file Data_Sheet_8.pdf]

Table 4 S

Data Quality for ***Media Sources****: Usage and Distress. Parents' and Children's Responses*

Media Source	N Valid (P/C)	% Missing (P/C)	% Zeros (P/C)	Gender Test *p* (P/C)	Geography Test *p* (P/C)
Instagram (Usage)	—/273	—/10.8%	—/35.9%	—/.359	—/.022
Instagram (Distress)	201/191 (165 pairs)	34.3%/37.6%	29.7%/45.5%	.863/.220	.022/.011
TikTok (Usage)	—/270	—/11.8%	—/34.4%	—/.714	—/.007
TikTok (Distress)	203/182 (160 pairs)	33.7%/40.5%	26.2%/45.6%	.607/.261	.003/.010
Facebook (Usage)	—/240	—/21.6%	—/69.6%	—/.395	—/.630
Facebook (Distress)	142/128 (99 pairs)	53.6%/58.2%	44.4%/59.6%	.300/.211	.286/.410
X (Twitter) (Usage)	—/228	—/25.5%	—/83.3%	—/.122	—/.865
X (Twitter) (Distress)	107/108 (75 pairs)	65.0%/64.7%	61.3%/72.0%	.195/.144	.503/.492
Telegram (Usage)	—/242	—/20.9%	—/59.9%	—/.045	—/.401
Telegram (Distress)	132/131 (98 pairs)	56.9%/57.2%	44.9%/51.0%	.228/.039	.005/.111
Other Social Media (Usage)	—/229	—/25.2%	—/67.2%	—/.246	—/.792
Other Social Media (Distress)	131/137 (88 pairs)	57.2%/55.2%	51.1%/55.7%	.738/.354	.858/.227
Television (Usage)	—/274	—/10.5%	—/21.5%	—/.559	—/.657
Television (Distress)	223/198 (178 pairs)	27.1%/35.3%	25.8%/34.3%	.133/.193	.002/.003
Printed Media (Usage)	—/236	—/22.9%	—/71.6%	—/.414	—/.979
Printed Media (Distress)	136/131 (98 pairs)	55.6%/57.2%	56.1%/58.2%	.071/.254	.008/.038
Online Newspapers (Usage)	—/235	—/23.2%	—/60.4%	—/.078	—/.977
Online Newspapers (Distress)	150/134 (102 pairs)	51.0%/56.2%	43.1%/52.9%	.417/.567	.612/.581
Conversation with Parents (Usage)	—/283	—/7.5%	—/7.4%	—/.335	—/.384
Conversation with Parents (Distress)	251/226 (204 pairs)	18.0%/26.1%	35.3%/41.7%	.603/.459	.189/.728
Conversation with Other Adults (Usage)	—/260	—/15.0%	—/35.4%	—/.281	—/.887
Conversation with Other Adults (Distress)	222/201 (167 pairs)	27.5%/34.3%	38.3%/47.3%	.785/.803	.039/.072
Conversation with Peers (Usage)	—/285	—/6.9%	—/7.0%	—/.924	—/.119
Conversation with Peers (Distress)	264/229 (215 pairs)	13.7%/25.2%	25.6%/36.3%	.847/.869	.352/.315

**Media Source**	**N Valid (P/C)**	**% Missing (P/C)**	**% Zeros (P/C)**	**Gender Test *p* (P/C)**	**Geography Test *p* (P/C)**
School Classes (Usage)	—/273	—/10.8%	—/19.8%	—/.132	—/.555
School Classes (Distress)	255/215 (196 pairs)	16.7%/29.7%	33.7%/46.4%	.551/.768	.031/.853

Note.
P = Parent; C = Child. Values before the slash represent parent data, values after the slash represent child data. Em dashes (—) indicate data not collected for that group. Usage measured in children only. Distress measured in parent-child pairs. *p*-values shown for gender and geography chi-square tests.
