# Supplementary material for: Children’s rights and needs during war: the case of adolescents in Israel
Source: Front Psychol. 2026 Mar 2;17:1719621. doi: 10.3389/fpsyg.2026.1719621 (PMC12989495; doi:10.3389/fpsyg.2026.1719621)
Supplement: Supplementary file 9 [file Data_Sheet_9.pdf]

Table S4-a

***Media Sources** Data Quality: Arabic and Hebrew Survey Sample Comparisons*

Variable	N Valid (A/H)	Missing N (A/H)	Missing % (A/H)	Zero % (A/H)
Instagram - Usage	17/92	7/9	29.2%/8.9%	11.8%/43.5%
Instagram - Distress	17/58	7/43	29.2%/42.6%	17.6%/29.3%
TikTok - Usage	16/90	8/11	33.3%/10.9%	25%/41.1%
TikTok - Distress	16/56	8/45	33.3%/44.6%	31.2%/39.3%
Facebook - Usage	14/78	10/23	41.7%/22.8%	64.3%/73.1%
Facebook - Distress	11/38	13/63	54.2%/62.4%	54.5%/76.3%
Twitter/X - Usage	14/78	10/23	41.7%/22.8%	78.6%/87.2%
Twitter/X - Distress	9/32	15/69	62.5%/68.3%	66.7%/78.1%
Telegram - Usage	14/77	10/24	41.7%/23.8%	42.9%/67.5%
Telegram - Distress	10/40	14/61	58.3%/60.4%	50%/62.5%
Other Social Media - Usage	12/80	12/21	50%/20.8%	58.3%/65%
Other Social Media - Distress	12/46	12/55	50%/54.5%	50%/58.7%
Television - Usage	16/85	8/16	33.3%/15.8%	12.5%/32.9%
Television - Distress	16/61	8/40	33.3%/39.6%	18.8%/39.3%
Printed Media - Usage	12/77	12/24	50%/23.8%	66.7%/61%
Printed Media - Distress	10/52	14/49	58.3%/48.5%	70%/61.5%
Online Newspaper - Usage	15/83	9/18	37.5%/17.8%	20%/61.4%
Online Newspaper - Distress	16/50	8/51	33.3%/50.5%	37.5%/58%
Conversation with Parents - Usage	17/91	7/10	29.2%/9.9%	5.9%/6.6%
Conversation with Parents - Distress	14/82	10/19	41.7%/18.8%	7.1%/47.6%
Conversation with Other Adults - Usage	16/85	8/16	33.3%/15.8%	18.8%/32.9%
Conversation with Other Adults - Distress	14/69	10/32	41.7%/31.7%	28.6%/49.3%
Conversation with Peers - Usage	14/91	10/10	41.7%/9.9%	21.4%/7.7%
Conversation with Peers - Distress	13/79	11/22	45.8%/21.8%	38.5%/38%
School Classes - Usage	12/85	12/16	50%/15.8%	58.3%/23.5%
School Classes - Distress	12/70	12/31	50%/30.7%	41.7%/54.3%

Note.
A = Arabic survey sample; H = Hebrew survey sample. Values before the slash represent Arabic survey sample data, values after the slash represent Hebrew survey sample data. Usage measured in children only. Distress measured in parent-child pairs. Missing N calculated as Total N minus N Valid. Zero % calculated from valid N only.
