# Supplementary material for: Children’s rights and needs during war: the case of adolescents in Israel
Source: Front Psychol. 2026 Mar 2;17:1719621. doi: 10.3389/fpsyg.2026.1719621 (PMC12989495; doi:10.3389/fpsyg.2026.1719621)
Supplement: Supplementary file 10 [file Data_Sheet_10.pdf]

Appendix Text A1: Empirical Justification for 60% Zero-Inflation Threshold

To establish an empirically justified threshold for zero-inflation, we examined distributional properties of all 127 continuous variables across Protection, Provision, and Participation domains in Wave I. After excluding 6 binary variables (education Yes/No items), we calculated skewness and coefficient of variation (CV) for each variable with ≥10 valid responses. Skewness measures distributional asymmetry, with values >2.0 indicating severe right-skewing where data function as quasi-binary indicators rather than continuous measures. CV (SD/Mean) quantifies relative variability, with values >1.5 indicating unstable variance typical of zero-inflated distributions. Variables were categorized as high (≥60% zeros) or low (<60% zeros) zero-inflation, and distributional metrics were compared between groups to validate the 60% threshold.

Results demonstrated clear distributional separation. Variables with ≥60% zeros (N=57, 47%) exhibited severe distributional violations: skewness M=3.65 (SD=2.26) and CV M=3.30 (SD=1.93). In contrast, variables with <60% zeros (N=64, 53%) showed near-symmetric distributions with stable variance: skewness M=0.14 (SD=0.83) and CV M=0.72 (SD=0.42). The 60% threshold effectively discriminated between variables unsuitable for statistical testing.
